# Single-cell transcriptional dynamics in a living vertebrate

**DOI:** 10.1101/2024.01.03.574108

**Published:** 2024-01-04

**Authors:** Elizabeth Eck, Bruno Moretti, Brandon H. Schlomann, Jordão Bragantini, Merlin Lange, Xiang Zhao, Shruthi VijayKumar, Guillaume Valentin, Cristina Loureiro, Daniele Soroldoni, Loïc A. Royer, Andrew C. Oates, Hernan G. Garcia

**Affiliations:** 1. Biophysics Graduate Group, University of California at Berkeley, Berkeley, USA; 2. Department of Molecular and Cell Biology, University of California, Berkeley, CA, USA; 3. Department of Physics, University of California, Berkeley, CA, USA; 4. Institute for Quantitative Biosciences-QB3, University of California, Berkeley, CA, USA; 5. Chan Zuckerberg Biohub – San Francisco, San Francisco, CA, USA; 6. Institute of Bioengineering, EPFL; Lausanne, CH; 7. Department of Cell and Developmental Biology, UCL; London, UK; 8. The Francis Crick Institute; London, UK

## Abstract

The ability to quantify transcriptional dynamics in individual cells via live imaging has revolutionized our understanding of gene regulation. However, such measurements are lacking in the context of vertebrate embryos. We addressed this deficit by applying MS2-MCP mRNA labeling to the quantification of transcription in zebrafish, a model vertebrate. We developed a platform of transgenic organisms, light sheet fluorescence microscopy, and optimized image analysis that enables visualization and quantification of MS2 reporters. We used these tools to obtain the first single-cell, real-time measurements of transcriptional dynamics of the segmentation clock. Our measurements challenge the traditional view of smooth clock oscillations and instead suggest a model of discrete transcriptional bursts that are organized in space and time. Together, these results highlight how measuring single-cell transcriptional activity can reveal unexpected features of gene regulation and how this data can fuel the dialogue between theory and experiment.

## Introduction

Development is driven by highly dynamic and coordinated gene regulatory programs. Rapid switches (Afzal and Krumlauf 2022), transient excursions (Bothma et al. 2014), and oscillations (Kageyama et al. 2008; Palmeirim et al. 1997) in gene expression are commonplace across the tree of life. While the last few years have resulted in an explosion of our ability to map the gene regulatory networks dictating developmental dynamics, predictively understanding the links between transcriptional dynamics, cell fate decisions, and tissue patterning remains an open, fundamental problem in developmental biology (Garcia et al. 2020).

**Figure 1:**
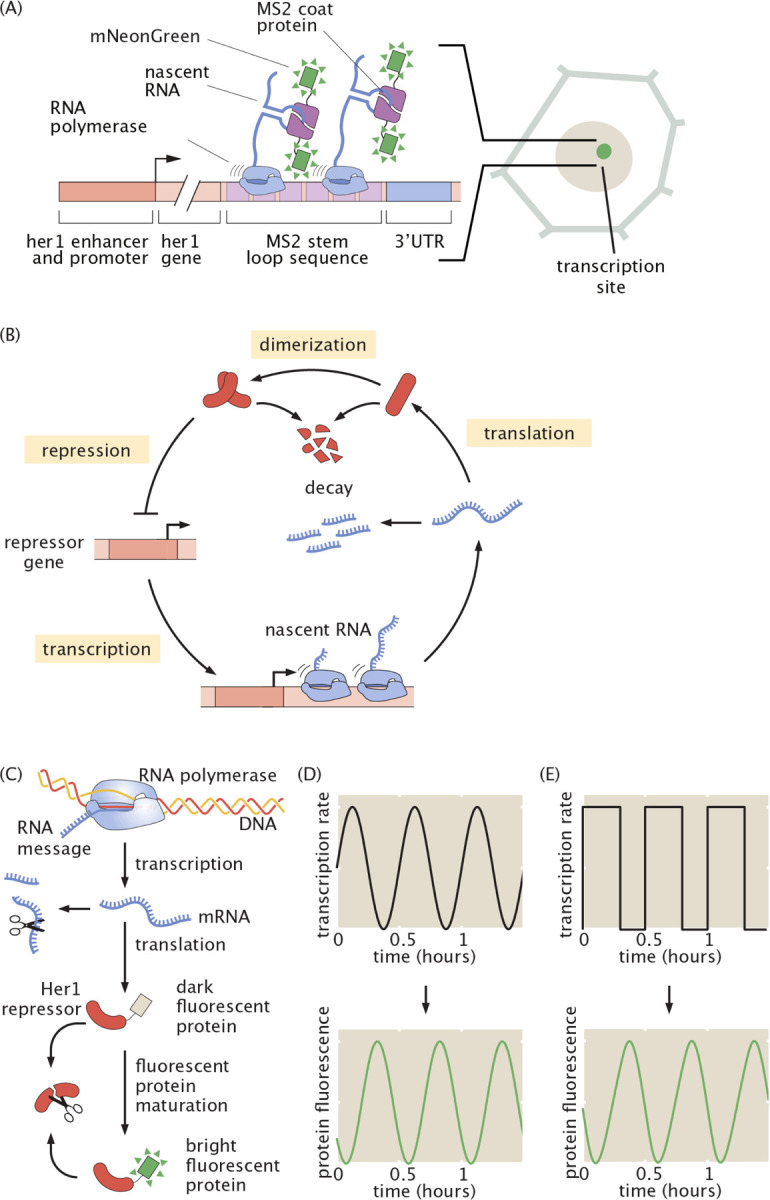
Protein reporters cannot distinguish between continuous and discrete modes of transcriptional regulation of the segmentation clock. (A) Schematic of the MS2 system, in which pre-formed fluorescent proteins (mNeonGreen) bind to nascent mRNA strands via a step loop-coat protein interaction. Fluorescence therefore accumulates at a site of nascent transcript formation. In this way, the MS2 system gives a near-real time readout of transcriptional activity.(B) Oscillations are generated via auto-repression. (C) Schematic of the central dogma showing the steps that lead to low-pass filtering. (D)-(E) Smooth protein oscillations can be generated either by smooth modulation of transcription rate (D) or discrete pulses (E), due to the low-pass filtering effect of product accumulation and decay.

The MS2-MCP system is a widely used method for precise, quantitative measurements of real-time transcriptional dynamics of individual genes (Bertrand et al. 1998; Golding et al. 2005; Chao et al. 2008; Larson et al. 2011; Garcia et al. 2013) (Fig. 1A). In this system, a transgene is tagged with multiple copies of a viral stem loop sequence (“MS2”), whose cognate binding partner (“MCP”, MS2 coat protein) is fused to a fluorescent protein. Upon mRNA synthesis, MCP binds the MS2 loops within the nascent transcript, producing a region of enhanced fluorophore concentration (a “spot”) that can be detected with a fluorescence microscope (Fig. 1A). The intensity of the resulting fluorescent spot is proportional to the number of RNA polymerase II molecules on the gene.

To date, precise, quantitative measurements of transcriptional dynamics via the MS2-MCP system have been limited to single cells in culture, tissue samples, invertebrate model organisms, such as flies and worms, and plants (Garcia et al. 2013; Lammers, Kim, et al. 2020; Wildner et al. 2023; Lee, Shin, and Kimble 2019; Corrigan et al. 2016; Alamos et al. 2021; Park et al. 2014). In these contexts, the MS2-MCP system has transformed our understanding of gene expression by revealing highly dynamic gene regulatory programs and novel molecular mechanisms of transcriptional regulation (Lim 2018). However, similar measurements have yet to be achieved in a living vertebrate. Given its established genetic toolkit and amenability to live imaging, the zebrafish is a prime candidate for exploring single-cell transcriptional dynamics in vertebrates. While MS2 has been used in zebrafish to study mRNA localization, previous implementations have faced issues of MCP aggregation that prevented quantification of transcription (Campbell et al. 2015; Fedder-Semmes and Appel 2021; Costa et al. 2020; Zhang et al. 2020).

Further, even if the genetic implementation of MS2 in vertebrates such as zebrafish was optimized, large challenges to using this technology to quantify transcriptional dynamics in intact vertebrate animals remain. For example, compared to flies or worms, zebrafish embryos are larger, denser, and exhibit greater motion during development, posing challenges in both image acquisition and analysis. Due to high cell motility during tailbud elongation (50 μm/h is a typical speed; see (Lawton et al. 2013)), fields of view hundreds of micrometers in length are required to track groups of cells for multiple hours. Simultaneously, one must be able to detect signals coming from diffraction-limited spots made of only tens of fluorophores. Large fields of view, over time, result in large (here, ~1 TB) datasets. The large size of the data, together with dense nuclei and strong, heterogeneous fluorescence backgrounds, complicate cell tracking and MS2 spot quantification. Thus, the full power of the MS2 system to quantify transcriptional dynamics has yet to be realized in zebrafish or any other vertebrate animal. As many aspects of vertebrate development and physiology fundamentally differ from invertebrates, entire fields of questions concerning transcriptional dynamics—including questions of direct biomedical relevance—remain unanswerable.

Here, we report the establishment of the MS2 system in transgenic zebrafish together with the development of an allied platform of light sheet fluorescence microscopy and computational image analysis to quantify transcriptional dynamics of MS2 reporters in complex and dynamical vertebrate embryos. We generated a new transgenic zebrafish line expressing a reengineered MCP that avoids protein aggregation. To enable the fast, large volume imaging, we used light sheet fluorescence microscopy and demonstrated that this method can retain the sensitivity required to detect MS2 spots. To make it possible to track cells and quantify spots in large datasets with strong background, we developed a computational image analysis pipeline that combines parallelized chunk-based file handling, GPU-acceleration, and deep learning, achieving fast and accurate measurements of transcriptional dynamics in single cells.

We applied this platform to a paradigmatic example of vertebrate-specific gene expression dynamics: the segmentation clock. In vertebrate embryo development, somites—morphological segments that prefigure the bones and muscles of the adult—are formed rhythmically and sequentially at the posterior end of the elongating body axis from the presomitic mesoderm (A. C. Oates, Morelli, and Ares 2012; Palmeirim et al. 1997). This rhythmic specification is dictated by a highly conserved biological clock that consists of an oscillatory gene regulatory network (A. C. Oates, Morelli, and Ares 2012), which is rooted in an auto-repression motif (Fig. 1B). While protein reporters have given insight into the dynamics of these oscillators, revealing smooth, sinusoidal oscillations in single cells, how oscillations are generated at the transcriptional level has been unknown. Due to the low-pass filtering effect of RNA and protein accumulation and degradation (Fig. 1C), high frequency dynamics are smoothed out at the protein level, making them invisible to the protein reporters available to date. For example, single-cell transcription rates could mirror protein levels, oscillating in a smooth, sinusoidal fashion (Fig. 1D), or they could be sharp and discrete (Fig. 1E). Both scenarios would produce smooth protein oscillations.

At the tissue scale, our transcriptional measurements recapitulate the known global picture of somitogenesis: the core clock gene *her1* expression oscillates over time and travels in a wave-like pattern from the tailbud to the forming somites. However, at the single-cell level we find that, in contrast to the smooth cycles of protein levels observed with fluorescent protein fusions, transcription rate is modulated in a sharp, discrete manner, resulting in bursts of transcription. While these bursts are largely periodic, they also exhibit a degree of stochasticity. Simulations of the protein oscillations predicted to occur based on these transcriptional dynamics are regular and sinusoidal, indicating a mechanism of robust oscillations that is compatible with stochastic transcription. To begin to explore how regular oscillations arise from transcriptional bursting, we developed a minimal mathematical model that extends the canonical two-state bursting model (Lammers, Kim, et al. 2020) to include auto-repressive feedback. A preliminary analysis of waiting time distributions for the active and inactive transcriptional intervals provides evidence for a mechanism in which Her1 dimers increase the probability of a burst ending, but where burst initiation events are purely stochastic and not affected by Her1 concentration.

All together, this work provides the tools necessary for studying transcriptional dynamics in zebrafish to the greater community and presents new measurements that challenge the existing paradigm of somitogenesis. Further, the pipeline presented here constitutes a framework that can be used to launch quantitative live imaging and theoretical dissection of transcriptional dynamics in other vertebrate systems.

## Results

### Implementing MS2 in zebrafish embryos to quantify transcriptional dynamics of the segmentation clock

**Figure 2:**
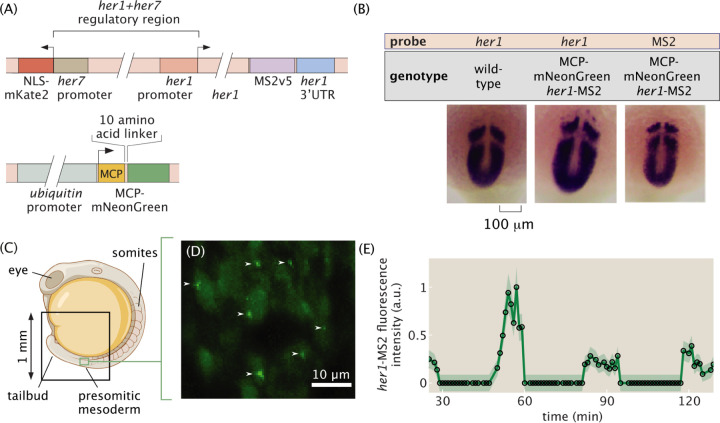
The MS2 system can be used to visualize transcriptional dynamics of the zebrafish segmentation clock. (A) Details of the transgenic MS2 and MCP constructs. Stable transgenic lines were made using I-SceI meganuclease transgenesis. (B) *In situ* hybridization assays comparing *her1* expression in wild-type and our transgenic embryos show that normal *her1* expression patterns are not affected by the presence of the *her1*-MS2 construct. Further, an *in situ* assay against MS2 shows that this reporter recapitulates endogenous *her1* expression (see Supplemental Fig. 1 for additional data). (C) Geometry of a zebrafish embryo. Black box represents the approximate field of view of the imaging experiment. Green box represents the approximate field of view of the zoomed-in area shown in (D). (D) Maximum intensity projection over 50 micrometers of the signal from MCP-mNG, *her1*-MS2 zebrafish showing several fluorescent spots (white arrows) against the hazy background MCP expression in individual nuclei. (E) Example *her1*-MS2 trace obtained for an individual cell within the presomitic mesoderm showing oscillations. Error bars reflect uncertainty due to background fluctuations and spot detection (see Methods). Panel (C) is adapted from BioRender.

While transient expression of transgenes via injection of one-cell stage embryos is common in zebrafish (Campbell et al. 2015), this approach is not ideal for reproducible, quantitative measurements of transcription due to the possibility of positional effects and other sources of variation from injections (Stuart et al. 1990; Roberts et al. 2014). Therefore, we generated two stable transgenic zebrafish lines via I-SceI meganuclease transgenesis (Soroldoni, Hogan, and Oates 2009): one carrying a reporter construct containing MS2 stem loop sequences inserted downstream of the *her1* regulatory region, dubbed “*her1*-MS2”, and another carrying an MCP-mNeonGreen (herein “MCP”) fusion driven by a ubiquitin promoter (Fig. 2A, Methods). The *her1*-MS2 construct was built off of previously established fusion protein reporter constructs, with the fluorescent protein switched for the MS2 stem loops (Soroldoni et al. 2014). Our approach differs from previous works on the MS2 system in zebrafish, which used transient injection of MS2 loops (Campbell et al. 2015; Fedder-Semmes and Appel 2021; Costa et al. 2020; Zhang et al. 2020).

We also made modifications to the MCP construct. Specifically, previous work used tandem dimer MCP with a nuclear localization signal (Campbell et al. 2015) and saw unwanted aggregation of the coat protein. We suspected that this aggregation was due to tandem dimer MCP and therefore used regular MCP. Further, while the nuclear localization signal has proven useful for keeping background levels low when monitoring transcript localization in the cytoplasm (Forrest and Gavis 2003), it can lead to overly high background levels in the nucleus that interfere with transcription measurements. Therefore, we did not include a nuclear localization signal in our construct.

We used I-SceI transgenesis because it is highly efficient and typically generates single insertion events (Soroldoni, Hogan, and Oates 2009). Single insertion events are ideal here because multiple insertion events could result in multiple MS2 spots, which would complicate our analyses. However, I-SceI transgenesis can insert multiple copies of the transgene at the same site, which raises two potential issues. First, multiple copies of the *her1* promoter might activate asynchronously, which would produce complicated MS2 signals. As we demonstrate below, we observe largely periodic signals, indicating either a single insertion or multiple insertions acting in unison. We elaborate on this point further in the Discussion section.

A second potential issue is that since our construct contains the full *her1* coding sequence, it is possible that extra functional copies of Her1 would alter clock function and produce developmental defects. To address this potential issue, we performed extensive characterization of segmentation clock patterning and somite formation in our transgenic lines. For both MS2 and MCP zebrafish lines, heterozygous animals showed normal patterns of *her1* expression (Fig. 2B, left and middle; Supplemental Fig. 1A–D) and minimal somite boundary formation defects consistent with previous Her1 protein reporter lines (Soroldoni et al. 2014; Delaune et al. 2012) (Supplemental Fig. 1F), indicating healthy somitogenesis. Further, in situ hybridization against the MS2 sequence mirrored the patterns of *her1* expression (Fig. 2B, right; Supplemental Fig. 1E), demonstrating that our construct faithfully reports on *her1* activity.

To assess the feasibility of detecting *her1*-MS2 spots, we imaged embryos on multiple types of fluorescence microscopes. To visualize nuclei, we crossed MCP-mNeonGreen fish with a line carrying h2b-mScarlet (O’Brown, Megason, and Gu 2019) (Methods). In *her1*-MS2/+; MCP-mNeonGreen/+; h2b-mScarlet/+ animals, green fluorescent puncta representing nascent *her1*-MS2 transcripts were readily detectable using both light sheet (Fig. 2C–D, Supplemental Fig. 2A–B) and laser-scanning confocal (Supplemental Fig. 2C–D) fluorescence microscopes. In control experiments with embryos expressing only MCP-mNeonGreen and no *her1*-MS2, fluorescent puncta were not detected (Supplemental Fig. 2B).

In preliminary time lapse imaging experiments on a laser scanning confocal (Zeiss 880 with AiryScan), we found that achieving even a barely-detectable MS2 signal with 1 minute time resolution (required to capture substructure within the oscillations, whose period is ~30 min) resulted in a small field of view of 100×50×20 μm^3^, or approximately 50 nuclei. To image the full tissue would require a field of view of approximately 500×500×400 μm^3^. Due to significant cell motion during somitogenesis (Lawton et al. 2013), we found that manual adjustment of the microscope stage was required every few minutes to keep the same cells in the field of view (Movie 1 ). To capture multiple oscillations, this constant adjustment must be done for multiple hours and the resulting movies registered together, making this approach impractical. Consequently, we exclusively used light sheet fluorescence microscopy, which enabled us to capture the full tissue containing somites, presomitic mesoderm, and tailbud, with 1 minute time resolution and acceptable signal-to-noise ratios (Royer et al. 2016) (Methods).

We built a computational image analysis pipeline (Supplemental Fig. 3A, B) to extract quantitative measurements of transcriptional dynamics from our images. As elaborated in the Methods section, the large size (~1 TB) of the image datasets and strong and heterogeneous background signal posed new challenges to the analysis of MS2 data. Specifically, the large size of the data called for out-of-memory computing schemes and speed optimization of every step of the pipeline, while the strong background required sophisticated algorithms for spot classification and localization. Overall, these challenges, which were new to the analysis of MS2 data in general, necessitated the development of specialized software. The pipeline tracks nuclei and identifies spots in parallel, then spots are assigned to nuclei and the sequences of MS2 spot intensities over time—”traces”—are constructed. Initial examination of traces revealed clear oscillations (Fig. 2E).

We performed extensive characterization of the pipeline’s accuracy (Methods, Supplemental Fig. 3C–H). Through comparison with 26 manually obtained traces, we found that our 3D nuclear tracking was approximately 80% accurate (21/26 nuclear trajectories showed no errors), with an error rate of around 1 nuclear tracking error per 5 nuclear trajectories, or on average 1 error per 500 minutes (see also Movie 2). Examples of tracking errors include swapping nuclear identities between neighboring cells, grouping two nuclei into one, and splitting one nucleus into two (Supplemental Fig. 3G).

Through comparison with manually curated traces, where spot detection was done entirely by human visual inspection, we found that spot detection was extremely accurate. Specifically, we found a false positive rate of 4% and a false negative rate of 8% (Supplemental Fig. 3H, I). When both manual traces and pipeline traces detected a spot, the resulting spot intensities were highly correlated with an *R*^2^ of 0.91 (Supplemental Fig. 3I). Through analysis of simulated spots, we found that our spot fluorescence quantification algorithm was also highly accurate, with typical errors of around 5% (Supplemental Fig. 4, Methods). As our MCP background was expressed quite heterogeneously throughout the tissue, it is possible that in some cells MCP levels were low enough to not saturate the MS2 stem loops, introducing a background-dependent signal (Wu, Chao, and Singer 2012). However, we observed only a very weak correlation (*R*^2^=0.26) between a spot’s background and its (background-subtracted) signal (Supplemental Fig. 3E), indicative of MCP saturation and reliable measurements of absolute fluorescence intensity.

Taken together, these results demonstrate that we successfully implemented the MS2 system in zebrafish for quantifying *her1* transcriptional dynamics. We then turned to studying the behavior of *her1* transcription at tissue and single-cell scales.

### *her1* transcriptional dynamics recapitulate protein oscillations and wave patterns at the tissue scale

We first assessed how the tissue-scale dynamics of the segmentation clock at the transcriptional level compare to the measurements obtained from Her1 protein dynamics reported by fluorescent protein fusions (Soroldoni et al. 2014; Delaune et al. 2012). Previous protein measurements revealed collective oscillations that travel as a phase wave from posterior to anterior, arresting at the formation of a new somite (Soroldoni et al. 2014; Delaune et al. 2012). The oscillation period varies with temperature, being approximately 30 minutes at 28 C.

**Figure 3:**
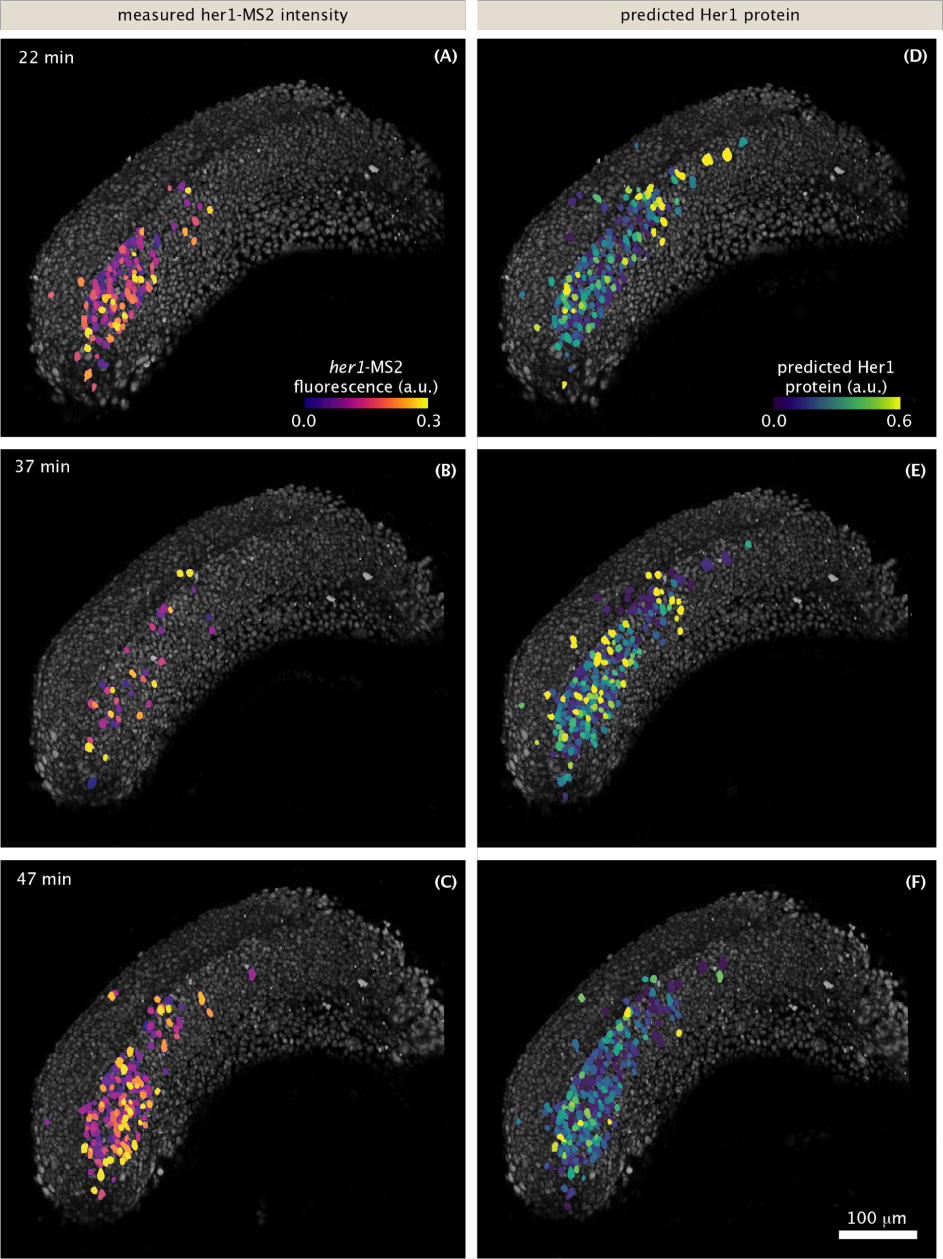
Transcriptional dynamics at the tissue scale recapitulate known features of protein patterns. Snapshots of 3D renderings of the presomitic mesoderm and tailbud during somitogenesis. (A-C): nuclei (gray) are false-colored in proportion to the intensity of their MS2 spots. (D-F): same as (A-C) but nuclei are colored in proportion to the predicted Her1 protein level, given the MS2 signal on (A-C) (Methods). See also Movie 3, Movie 4, and Movie 5. Scale bar: 100 μm. Time is measured from the start of image acquisition, which corresponds to approximately the 15 somite stage.

While the dynamics of protein reporters can be directly visualized in movies (Soroldoni et al. 2014; Delaune et al. 2012), MS2 spots are too small and dim to be picked up in raw 3D renderings or projections of the full tissue. Therefore, to visualize transcriptional dynamics, we false colored nuclei in proportion to their spot intensity (Fig. 3A–C, Movie 3, Movie 4). The resulting movies showed oscillations and waves similar to movies of fluorescent proteins, qualitatively recapitulating previous protein-level dynamic measurements.

However, some differences between our transcriptional movie and previous protein movies are noticeable. First, the bulk wave patterns we observe are noisier, with a higher prevalence of high frequency flashing. Second, there are clear periods of relative silence tissue-wide (compare Fig. 3B to Figs. 3A and 3C, Supplemental Fig. 5) that are not apparent at the protein level.

To more directly validate our construct against previous protein measurements, we compared the protein dynamics predicted by our transcriptional data to actual protein-level measurements of Her1 dynamics. Using a linear production/decay model (based on Fig. 1C) and our MS2 traces as input, we generated visualizations of the predicted Her1 protein concentrations (Fig. 3D–F, Movie 5). These predicted protein patterns are smoother than the MS2 patterns, showing gradual waves traveling from posterior to anterior. These patterns are visually similar to previous movies of actual protein reporters (Soroldoni et al. 2014; Delaune et al. 2012). The silent periods observed in the MS2 data were largely absent in the predicted protein data, consistent with previously measured protein dynamics (Soroldoni et al. 2014; Delaune et al. 2012). The absence of silent periods in protein dynamics despite the existence of such silent periods in transcription is due to the added timescales of mRNA and protein decay (on the order of 10 minutes each) smoothing and delaying the oscillations. Thus, once protein production and decay are taken into account, our transcriptional reporter faithfully recapitulates the behavior of established protein reporters at a qualitative level.

**Figure 4:**
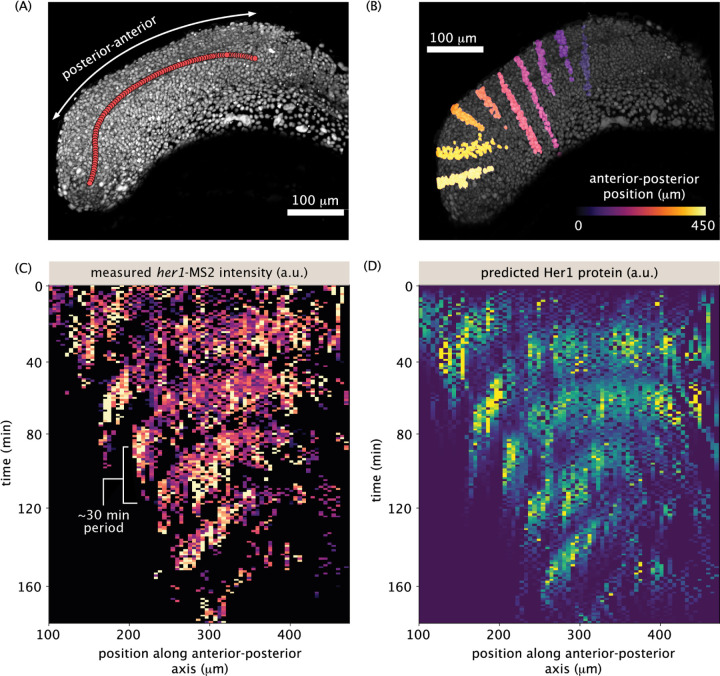
Tissue-scale analysis reveals transcriptional oscillations and wave-like patterns. (A) 3D rendering of nuclei (gray) illustrating the anterior-posterior (‘AP’) axis (red circles). The axis is defined coarsely every 50 time points and then spline interpolated in both space and time (Methods). See also Movie 3. (B) 3D rendering of nuclei (gray) illustrating example bins along the AP axis (colored nuclei). For simplicity, every tenth bin is highlighted. (C-D) Kymographs of total *her1*-MS2 (C) spot intensity and predicted Her1 protein (D). Distance is defined along the anterior-posterior axis as in (A). Spot AP positions are binned, with bin size = 6.5 microns, and the sum of all intensities in each bin if plotted for each time point. In (C) and (D), contrast limits are set from 0 to 0.4 times the maximum bin intensity.

To quantitatively validate our reporter, we captured transcription and protein spatiotemporal patterns in kymographs (Fig. 4). To make this possible, we defined an anterior-posterior axis with a combination of manual labeling (following the notochord) and spline interpolation (Fig. 4A, Movie 6, Methods). We grouped cells into equally-spaced bins along this axis (Fig. 4B, every 10th bin is shown for visual clarity), summed the spot intensities in each bin and plotted them over time. The resulting MS2 kymograph (Fig. 4C) shows clear, regular oscillations throughout the tissue, with periods of approximately 30 min, consistent with protein measurements (Soroldoni et al. 2014). Further, the left-down sloping of the stripes indicates the traveling phase wave from posterior to anterior, with a speed of approximately 100 μm/h, consistent with previous measurements (Soroldoni et al. 2014). The predicted protein kymograph (Fig. 4D) largely mirrors the MS2 kymograph, though the protein kymograph is visibly smoother and phase shifted with respect to the MS2 one, as expected from the time lag between transcription and translation.

All together, these observations demonstrate that, at the tissue scale, the transcriptional dynamics of the segmentation clock largely mirror protein dynamics, with some differences: transcriptional activity appears noisier than accumulated protein levels, and the total amount of *her1* transcription clearly oscillates in time.

### Single-cell transcriptional oscillations comprise sharp, quasi-periodic bursts

**Figure 5:**
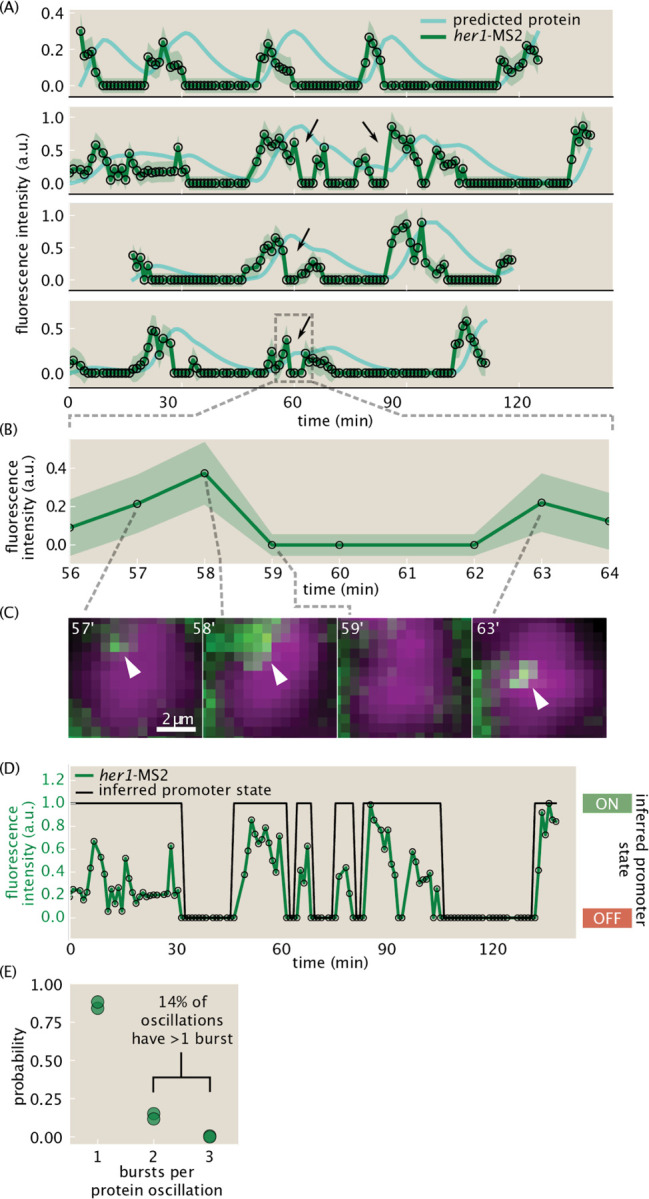
Single-cell traces reveal transcriptional bursts. (A) Measured single-cell *her1*-MS2 traces (green) and predicted Her1 protein levels (cyan) using a production/decay model (Methods). Examples of multiple bursts giving rise to a single predicted protein oscillation are highlighted with black arrows. Shaded error bars reflect uncertainty due to background fluctuations and spot detection (Methods). (B) Zoom in to one burst in the last trace of (A). (C) Single z-slices images showing examples of the rapid disappearance and reappearance of a *her1*-MS2 spot, corresponding to the burst highlighted in (B). The z-slice of minute 59 is the same as the previous time point. Spots are highlighted with white arrowheads. Green=her1-MS2; MCP-mNG. Magenta=h2b-mScarlet. (D) Example of promoter state inference via trace binarization. (E) Distribution of number of bursts per predicted protein oscillation for two embryos. 14±2% of oscillations have multiple bursts within them.

Exploiting the unique power of the MS2 system, we next investigated transcriptional dynamics in single cells. Individual MS2 traces (Fig. 5A, green lines; Supplemental Fig. 6) show clear periodicity. However, in contrast to previously measured smooth protein oscillations (Delaune et al. 2012; Soroldoni et al. 2014), our observed transcriptional dynamics are strikingly sharp and discrete. Further, we observed examples of multiple, distinct transcriptional pulses in rapid succession, which we refer to as bursts (Fig. 5A, black arrows). We visually inspected these bursts and confirmed the rapid disappearance and reappearance of the MS2 spot (Fig. 5B, C). Remarkably, even for these cases with a higher frequency of transcriptional bursting, predicted protein traces for single cells are smooth, roughly sinusoidal, and regular in period, similar to measured protein traces (Fig. 5A, cyan lines; Methods) (Delaune et al. 2012; Rohde et al. 2021).

To quantify the bursty character of our traces, we implemented a conservative burst-calling algorithm based on simple thresholding of a moving-averaged filtered trace that infers the underlying promoter state (Fig. 5D, Methods). With this approach, a pulse in MS2 signal is called a separate burst only if it is surrounded by at least 3 minutes of inactivity (no spots detected for 3 time points) on either side. Therefore, our analysis likely undercounts bursts and does not consider bursts that occur on timescales less than a few minutes.

Combining promoter state and predicted protein data, we measured that 14±2% (mean ± std. dev. across two embryos) of protein oscillations are generated by 2 or more transcriptional bursts (Fig. 5E, Methods). We validated this result on a subset of 28 manually curated traces, for which we visually confirmed that each drop in MS2 signal between bursts corresponds to a clear disappearance of a spot in the image and not to an error in spot detection or nuclear tracking. On this manually curated dataset, we measured that 19±7% of protein oscillations are generated by 2 or more transcriptional bursts, in agreement with the result obtained from the full dataset.

These sharp, noisy pulses are reminiscent of transcriptional bursts in other unicellular and multicellular systems (Lammers, Kim, et al. 2020; Tantale et al. 2016; Chubb et al. 2006; Muramoto et al. 2012; Fukaya, Lim, and Levine 2016; Lee, Shin, and Kimble 2019). Canonical bursts are stochastic (Lammers et al. 2020), at odds with the notion of regular oscillations. However, unlike canonical bursty systems, the segmentation clock is strongly regulated by auto-repression: Her1 proteins repress *her1* transcription (Fig. 1B). This feedback loop must produce or modulate bursts in such a way as to generate regular oscillations. To shed light on the mechanism by which Her1 regulates its own transcriptional bursting and how stochastic transcriptional bursts might interplay with auto-repression to generate noisy but regular oscillations, we turned to mathematical modeling.

### Burst timing statistics support a model of burst duration regulation

**Figure 6:**
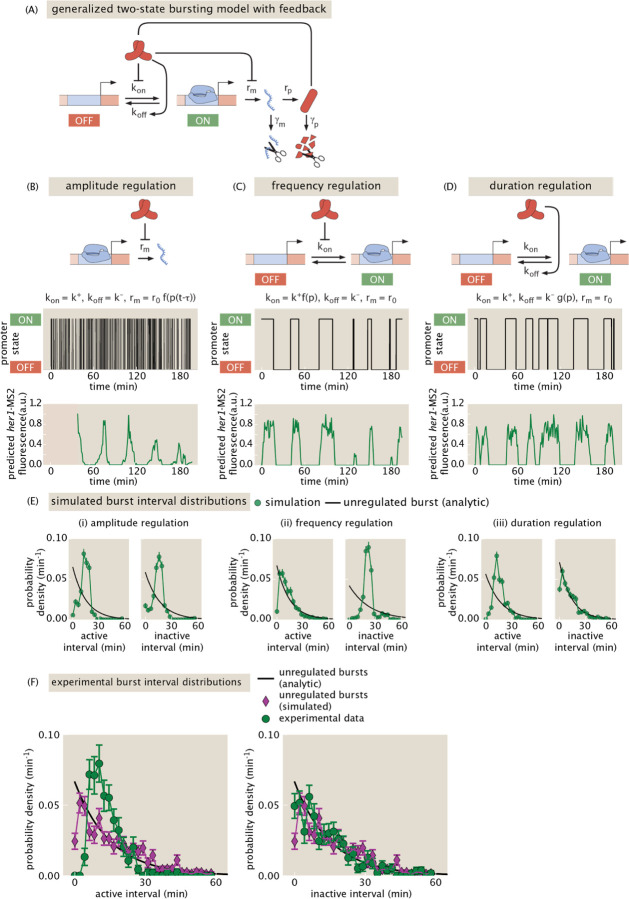
A two-state model of transcriptional bursting with feedback suggests multiple mechanisms of repression could generate oscillations. (A) Schematic of the bursting model. The promoter switches between active (ON) and inactive (OFF) states with rates *k*_*on*_ and *k*_*off*_. In the active state, mRNA is transcribed at rate *r*_*m*_ and then translated into protein. The protein can then modulate any of *k*_*on*_, *k*_*off*_, and *r*_*m*_ to repress mRNA production and generate oscillations. (B)-(D) Example of oscillations generated by modulation of the transcription rate, *r*_*m*_, (‘amplitude regulation’), *k*_*on*_, (‘frequency regulation), and *k*_*off*_ (duration regulation) respectively. Each panel shows the binary promoter state (top) and a simulated MS2 trace (bottom). See the Methods section for parameter values. (B) Amplitude regulation: the transcription rate is repressed by the protein according to a decreasing Hill function, *f(p)* evaluated at a delayed protein value p(t-*τ*). Because of this delay, the prediction of the MS2 signal before t=*τ* cannot be computed, as indicated by the red shaded region. (C) Frequency regulation: *k*_*on*_ is repressed according to the same decreasing Hill function *f*(*p*). No explicit delay is required to generate oscillations. (D) Duration regulation: *k*_*off*_ increases with protein level according to an increasing Hill function *g*(*p*). (E) Signatures of the three different regulatory strategies in the active and inactive interval distributions. Shown in green are model predictions for the active and inactive burst interval distributions under the three regulatory strategies, accounting for measurement noise, a detection threshold of MS2 signals, and a realistic number of data points. Black lines are maximum likelihood estimates of an exponential distribution, the theoretical expectation under a model of unregulated bursting. Under amplitude regulation, both active and inactive intervals are non-exponential. Under frequency regulation, active interval distribution is approximately exponential, with a cutoff at short intervals due to the detection threshold, but the inactive interval distribution is non-exponential. Under duration regulation, the active interval distribution is non-exponential, while the inactive distribution is well-described by an exponential distribution. Parameters are the same as in (B)-(D). (F) The experimentally measured interval distributions (green circles) support a model of duration regulation, with the active interval distribution being non-exponential and the inactive interval distribution being exponential. For comparison, overlaid are simulated distributions (magenta) from a model with purely unregulated bursts and realistic MS2 acquisition parameters that have the same means as their experimental counterparts (See the Methods section for details on MS2 signal simulations). Also overlaid are the ideal, analytic exponential distributions (black lines).

The ability to directly observe transcriptional bursting in the segmentation clock raises new possibilities for uncovering the molecular mechanisms underlying clock oscillations through an iterative dialog between experiments and theory. To begin to explore how bursting might interplay with regular oscillations, we turned to mathematical modeling (Fig. 6A). We extended the classic two-state bursting model to include feedback that accounts for the auto-repression of *her1*, ignoring interactions with other clock components such as *her7* (Appendix, Section 3). Feedback can occur via regulation of transcription rate, (‘amplitude regulation’), burst frequency (‘frequency regulation’), burst duration (‘duration regulation’), or a combination thereof. In preliminary explorations of parameter space, we found that quasi-regular oscillations can emerge from the sole regulation of either amplitude (Fig. 6B), frequency (Fig. 6C), or duration (Fig. 6D). This observation points to the possibility of dynamically rich regulatory strategies, where multiple aspects of bursts are modulated to achieve oscillations, and calls for theoretical predictions that can be tested to rule in or out each strategy.

As a first step in uncovering the mechanisms regulating *her1* bursts, we attempted to identify signatures of the different regulatory strategies of bursting dynamics in the distribution of active and inactive intervals of bursts. The active interval, also known as burst duration, is the length of time that the promoter is in the ON state. The inactive interval is the interval over which the promoter is in the OFF state. In unregulated bursting, the distributions of both intervals are exponential, reflecting the Poisson-nature of the ON and OFF switching events. At the opposite extreme, regular oscillations have narrow, peaked interval distributions that become more peaked as the oscillations become perfect. We reasoned that when *k*_*on*_ is regulated but not *k*_*off*_, the active interval (controlled by *k*_*off*_) should follow an exponential distribution while the inactive interval (controlled by *k*_*on*_) should be more peaked to allow for bursts to occur in a periodic fashion. Alternatively, for *k*_*off*_ regulation, we expect the inactive interval should be exponentially distributed, while the active interval would be peaked. To account for the MS2 measurement process affecting our ability to resolve bursts, we simulated realistic MS2 traces from the promoter state trajectories created by each model according to an established method (Lammers, Galstyan, et al. 2020), incorporating a finite sampling rate, measurement noise, and a detection threshold (Methods). We then applied the same burst calling algorithm on this simulated dataset as we used on the real data, and computed the distributions of active and inactive intervals.

For the amplitude regulation parameter set in Figure 6B that supports oscillations, we found that both active and inactive interval distributions were non-exponential. Instead, these distributions were peaked, consistent with regular transcriptional oscillations (Fig. 6E(i)). While both intervals should in theory follow an exponential distribution, in this example the bursting dynamics are faster than the experimental sampling time. As a result, individual bursts are blurred together into regular, composite bursts whose interval distributions are non-exponential. For frequency regulation, as expected, the inactive interval distribution was non-exponential, while the active interval distribution was largely exponential but with a short interval cutoff due to the shortest bursts producing MS2 signals below our detection limit (Fig. 6E(ii)). For duration regulation, the opposite held (Fig. 6E(iii)). These differing predictions for burst timing statistics offer a first pass for opportunities to uncover which regulatory strategies are at play.

Turning to the data, we found interval distributions most similar to those in our example of duration regulation—a peaked, non-exponential active interval distribution, (Fig. 6F, green circles) and a broader, exponential inactive interval distribution (Fig. 6G, green circles). To assess the significance of this deviation of the active interval distribution from an exponential, we compared the data to simulations of a null model of unregulated bursting, with *k*_*on*_ and *k*_*off*_ set by the average interval distributions in the experimental data (Fig. 6F, magenta diamonds). Even accounting for the MS2 measurement process, the measured distribution is more peaked than the unregulated null model, which closely aligns with the theoretical exponential distribution (Fig. 6F, black line). In contrast, the inactive interval distribution is indistinguishable from both simulated and analytic predicted unregulated distributions (Fig. 6G). This preliminary result supports a model in which the *her1* promoter stochastically fluctuates between ON and OFF states, but Her1 proteins increase the probability of switching to the OFF state in such a way as to create regular oscillations of Her1 protein. However, more work must be done to validate this result, including properly assessing the role of the experimental detection limit in altering the shape of these interval distributions, as well as more thoroughly exploring parameter space of the model.

While there is more work to do, we emphasize that these measurements of burst dynamics are the first of their kind in a living vertebrate and make possible a wide range of exciting directions. In particular, combining the MS2 technology and theoretical model developed here with perturbations that alter segmentation clock function will allow us to directly test the hypothesis of duration regulation.

## Discussion

In this work, we established the use of the MS2-MCP mRNA labeling system for quantifying transcriptional dynamics in zebrafish embryos, to our knowledge, a first in any intact vertebrate animal. We used this technology to measure the dynamics of *her1*, a genetic oscillator in the core of the segmentation clock. In contrast with the smooth, sinusoidal oscillations seen at the protein level (Delaune et al. 2012; Soroldoni et al. 2014), at the transcription level oscillations are discrete and burst-like. Due to the low-pass filtering effects of RNA and protein accumulation, these high frequency bursting dynamics get smoothed out at the protein level. Thus, our finding highlights the power of the MS2 system in being able to reveal novel dynamical features of gene regulation that would remain invisible to the classic approach of fusing proteins to fluorescent proteins.

Our observation of segmentation clock oscillations being composed of discrete transcriptional bursts raises the question of how these bursts are created and regulated to ensure proper protein dynamics that will ultimately drive vertebrate segmentation. In a simple mathematical model, we found that quasi-regular oscillations could be produced if Her1 protein modulated burst amplitude, frequency, or duration, or a combination thereof. We found preliminary evidence for a model of duration regulation in the form of burst timing distributions, echoing the results of similar, previous models (Wang, Lefranc, and Thommen 2014; Lengyel and Morelli 2017) (see Appendix, Section 3.5 for more discussion). The mechanism of duration regulation could be more directly validated by measuring *her1* transcription while knocking down Her1 protein, either through mutants or morpholinos, and looking for changes in burst amplitude, frequency, or duration. Such an experiment is only possible with RNA labeling technologies like MS2: as demonstrated above, these different mechanisms can give rise to identical protein dynamics and are only distinguishable at the level of transcriptional dynamics. Moreover, disentangling effects of breaking the feedback loop from effects of the knockdown itself using a Her1 protein reporter would be complicated because Her1 protein dynamics cannot be reliably quantified if the Her1 protein itself is knocked down (Andrew C. Oates and Ho 2002). Our tools thus present a unique opportunity to dissect the transcriptional regulation of an auto-repressive system.

One potential caveat to our interpretation of multiple bursting events during one protein oscillation is the possibility of multiple copies of the *her1*-MS2 reporter construct having been inserted during transgenesis. I-SceI meganuclease transgenesis typically produces only one integration event, but can integrate multiple copies at the same site (Soroldoni, Hogan, and Oates 2009). In this case, some of the stochasticity in bursting we observe could be due to the independent activation of multiple reporter copies, rather than to fluctuations of a single promoter’s state. The fact that the majority of *her1*-MS2 traces are regular, with one transcriptional burst per protein oscillation, suggests that, if multiple reporter copies exist, they act largely in unison. This interpretation is consistent with recent work on the pairing of *her1* and adjacent clock gene *her7*, which showed that when *her1* and *her7* are on the same chromosome (in *cis*) they are more likely to transcribe concurrently than when they are on opposite chromosomes (in *trans*). Future analysis of the nature of the insertion will be needed to clarify this issue. Specifically, new landing site technologies for the insertion of transgenes in a single copy would make it possible to circumvent any of the potentially confounding effects of multiple copy insertions (Mosimann et al. 2013; Bhatia et al. 2021; Hadzhiev et al. 2016).

Despite the potential for our fish containing multiple copies of the transgene, most of our results are largely insensitive to the reporter copy number. The tissue-scale dynamics of Figure 4C average over individual cells, washing out small-scale fluctuations. The relative stochasticity of active and inactive transcriptional intervals in Figure 6F would be sensitive to different copies of the promoter firing independently. However, the fraction of multiple burst events per protein oscillation is small (~14%). Therefore, even if we assume that all of these multiple burst events are the result of multiple copies of the reporter and exclude them, the overall shape of the active and inactive interval distributions would be unaffected. Finally, the measurement most affected by multiple copies would be the distribution of the number of bursts per protein oscillation (Fig. 5E).

One further limitation of our results is that, at present, it is unclear to what extent one can compare the amplitude of our MS2 signals from different nuclei. Systematic error in the overall fluorescence intensity of spots can arise from multiple sources, including cell-cell variability in MCP expression from our ubiquitin promoter, variable light scattering with tissue depth, and non-uniformity of the excitation beam in light sheet imaging. Exploring alternative promoters with less variability for driving MCP expression will be a useful future endeavor. Further, while the quantification of fluorescence with laser-scanning confocal microscopy is relatively well established, future work is needed to more rigorously assess how to maximize the accuracy of in vivo quantitative fluorescence measurements done with light sheet microscopy. Addressing these issues will be important for inferring the degree of burst amplitude regulation present in the segmentation clock, though should not affect inference of burst timing regulation.

Outside the segmentation clock, the role of transcriptional bursting in genetic oscillators is relevant to a wide range of phenomena. Interestingly, we are aware of two other examples of MS2 measurements of oscillating genes, both of which report bursts within protein-level oscillations. Hafner et al. measured transcription in the p53 pathway and found that approximately 30% of p21 oscillations (period ~5 hours) contained multiple bursts (Hafner et al. 2020). Wildner et al. studied transcription in the yeast gene CUP1 in response to metal stressors and inferred bursting on the timescale of minutes within oscillations of period ~30 minutes (Wildner et al. 2023). Together with our results, these findings suggest that transcriptional bursting may in fact be a common feature of genetic oscillators.

Beyond zebrafish, we expect several of the challenges faced here to also be present in other vertebrate systems, such as organoids and mouse pre-implantation embryos. The large size and photosensitivity of these systems make light sheet fluorescence microscopy a useful imaging approach, which comes with well-known challenges of image processing and analysis. Further, these tissues are also often dense and possess strong, structured fluorescence backgrounds that complicate particle detection (Fu et al. 2023). Our image analysis pipeline for MS2 spot quantification, along with the plasmids for MCP and MS2 constructs, may be a useful starting point for such endeavors.

In sum, we envision the tools presented here will be useful for expanding this field further into the domain of vertebrate tissues, where they will help us understand how highly dynamic gene expression programs achieve robustness, and how they fail to do so in disease states.

## Methods

### Ethics statement

All experiments with zebrafish were done in accordance with protocols approved by The University of California, Berkeley’s Animal Care and Use Committee and following standard protocols (protocol number 2018-01-10640-1). All zebrafish used in this study were embryos in the somitogenesis period. Sex differentiation occurs later in zebrafish development (Westerfield 2007) and thus was not a factor in our experiments.

### Zebrafish

Transgenic zebrafish lines of *her1*-MS2 and MCP-mNeonGreen (Fig. 2A) were generated using I-SceI meganuclease-mediated transgenesis (Soroldoni, Hogan, and Oates 2009). Briefly, DNA was co-injected with I-SceI meganuclease into the cell of one-cell stage wild-type embryos (AB for *her1*-MS2, TL for MCP-mNeonGreen). Post-injection, I-SceI enzyme activity was assayed by electrophoresis. One day later, embryos were screened to confirm the fluorescence of mKate2, mNeonGreen, or eGFP, whichever was appropriate to the injected DNA. Injected embryos were raised to adulthood and outcrossed to wild-type (AB or TL) zebrafish. If offspring exhibited fluorescence, they were selected to be raised for experiments and their parent was selected as a founder.

The MCP construct uses a ubiquitin promoter (Mosimann et al. 2011) and encodes a ten amino acid linker between the coat protein and fluorescent protein.

The MS2 reporter transgene uses the shared regulatory region between *her1* and *her7*, along with the *her1* coding sequence (Soroldoni et al. 2014). The *her7* coding sequence was replaced with nuclearly localized mKate2, a red fluorescent protein, which serves as a marker of transgenesis (and the presence of MS2) during embryo screening. The *her1* promoter drives the expression of the *her1* coding sequence followed by *MS2v5* (Wu et al. 2015) inserted just before the *her1* 3’UTR.

Sequences for all constructs used in this work can be found at https://benchling.com/garcialab/f_/f9Cist7M-zebrafish-ms2-paper/.

Zebrafish lines were created at the Max Planck Institute at Dresden and then transferred to the UC Berkeley Zebrafish facility, where they were raised with standard procedures.

For a nuclear marker, we used a line carrying h2b-mScarlet (O’Brown, Megason, and Gu 2019). MCP-mNeonGreen fish were crossed with h2b-mScarlet and offspring were screened for the presence of both fluorophores on a Zeiss AxioZoom widefield microscope.

For imaging, the combined MCP-mNG; h2b-mScarlet fish were crossed with *her1*-MS2 in the evening and embryos were transferred to 19°C after they reached shield stage. The next morning, embryos were screened for mNeonGreen and mScarlet, and transported from UC Berkeley to the CZ Biohub for light sheet imaging. The presence of the *her1*-MS2 reporter was screened visually on the light sheet via observation of spots.

### Light sheet imaging

Embryos were imaged using two microscopes: a Zeiss Z1 light sheet microscope and *Dorado*, a 4-objective multiview home-made light sheet microscope (Royer et al. 2016). For both microscopes, embryos were dechorionated using sharp forceps, embedded in 0.1% low melting point agarose (dissolved in E3 media), and pipetted inside optically clear FEP tubes (Zeus Inc. custom order, 2 mm inner diameter) following an established protocol (Kaufmann et al. 2012). E3 media was defined as 5mM NaCl, 0.17mM KCl, 0.33mM CaCl2, 0.33mM MgSO4, pH adjusted to 7.3 with Tris-HCl pH 7.5 1M. A plug of 1.5% agarose was inserted at the bottom of the tube to prevent the leakage of the lower-concentrated agarose. The embryos were oriented such that their tailbud pointed toward the wall of the tube. Once mounted on the microscope, the samples were excited with two light sheets using 488 nm and 560 nm lasers sequentially to avoid bleed-through.

#### Multiview light sheet details:

The light sheets were generated using two Nikon N10XW-PF objectives (0.3 NA, 3.5 mm WD). Stacks of 400 z-slices with a spacing of 0.97 μm and a frame interval of 1 minute were acquired during 3 hours at 28 C. The total size of the field of view was 388×1000×1000 μm (*z*-*y*-*x*), with a pixel size of 0.485 μm in *x-y*. Images were acquired using two Nikon N16XLWD-PF detection objectives (0.8 NA, 3.0 mm WD) and two Hamamatsu ORCA-Flash 4.0 V3 Digital CMOS cameras. The use of two lightsheets and two cameras results in four images per channel for each time point, which are later computationally fused as described below.

During image acquisition, we encountered a hardware communication issue that led to a blocked filter wheel and subsequently missed images for approximately 10% of time points. We removed these blank time points but accounted for the missing time intervals. The rest of the scans were unaffected.

#### Zeiss Z1 light sheet details:

The light sheets were generated using one Zeiss 10x/0.2 illumination objectives. Stacks of 409 z-slices with a spacing of 1 μm and a frame interval of 1.5 minutes were acquired over 3 hours at 25 C. The total size of the field of view was 409×437×437 μm (z-y-x), with a pixel size of 0.228 μm in *x-y*. Images were acquired using a Zeiss 20x/1.0 water immersion objective and two PCO Edge 5.5 CMOS cameras. In this experiment, only one light sheet and one camera per color channel were used, so no fusion of images from multiple cameras was required.

### Data and code availability

Code for the full image analysis pipeline can be found at https://github.com/bschloma/zms2.

The output of this code for the two datasets in this paper along with two manually curated datasets and additional files included as Supplementary Data Files.

Code for reproducing the plots in this paper, including model simulations, can be found at https://github.com/bschloma/zebrafish-ms2-paper.

### Image processing

The raw images were first converted to the *zarr* format (Miles et al. 2020). Then, for each time point, the four images that constitute each channel were fused using the *dexp* Python package (Yang et al. 2022). The fusion is done in two steps. First, the volumes from different light sheets and the same camera are fused, resulting in one volume per camera, two volumes in total. These two volumes are registered to compensate for a minimal misalignment between the cameras and then fused, resulting in a single volume. During fusion, the brightness of one of the volumes is adjusted to the other, and then, they are blended together. To increase the robustness of the registration, the median translation from multiple registrations between volumes from different cameras is used. After fusing the images, the intensity of the Histone channel was normalized across the time series. A Gaussian blur and background subtraction were also applied during this step to facilitate nuclear segmentation, but no further processing was done to the MCP channel.

### Nuclear segmentation and tracking

Cell nuclei and their boundaries were detected using a 3D U-NET (Ronneberger, Fischer, and Brox 2015) (code available upon request). Detected nuclei were segmented and tracked over time using the approach described in (Bragantini, Lange, and Royer 2023). Both the segmentation and tracking were performed on a desktop computer (Intel Core i9 11900K, GeForce RTX 3090 24GB, 128 GB RAM) running Ubuntu 20.04.

### Spot analysis

To perform spot analysis, we developed a custom Python pipeline. Our pipeline takes in two *zarr* arrays, one corresponding to the MCP channel images and the other corresponding to a label matrix that encodes the nuclear segmentation and tracking information, and outputs a *pandas* DataFrame with spot location and intensity information.

The goal of the spot analysis is to locate all of the true MS2 spots and quantify their intensity. The pipeline is organized into the following steps (Supplemental Figure 3A):

1. *Detection:* a first pass of detecting spots is performed using simple filtering and thresholding.
2. *Classification:* voxels containing detected spot-like objects are classified into ‘spot’ and ‘not spot’ using a convolutional neural network classifier, which outputs a probability of belonging to the ‘spot’ class. The user provides a threshold probability value to reject false spots (we used 0.7).
3. *Quantification:* the intensity of the remaining spots is measured by estimating a background level of pixels just outside the spot, subtracting this background from spot pixels, and summing the resulting pixel values.

#### Detection:

First, bright skin cells that surround the presomitic mesoderm were removed from the image using blurring and thresholding. Then, potential spots were identified with a simple Difference of Gaussians filter and thresholding the filtered image. The filtering and binary thresholding is done on each 2D z-slice independently on a GPU using the *cucim* library. The resulting 3D mask was then transferred back to the CPU for the label matrix calculation. The centroid of each object in the label matrix was computed as an approximate location of the potential spots in the image.

#### Classification:

A small convolutional neural network was trained to classify voxels as containing a spot or no spot. Voxels were 9×11×11 pixels^3^. The network was implemented in *Keras*. The network architecture was inspired by a model used to classify fluorescence microscopy images of bacteria (Hay and Parthasarathy 2018). Schematically, the network consists of 2 convolution layers followed by a fully connected layer that outputs the probability of containing a spot. Specifically, the first 3D convolutional layer has 4 kernels, each of size 3×3×3 pixels^3^, ReLU activation and ‘same’ padding, followed by max pooling. The second 3D convolutional layer has the same parameters as the first and is also followed by max pooling. We then flatten into a dense layer with 512 neurons and ReLU activation. During training, this layer is followed by a Dropout layer with dropout = 0.5. The result is then fed into a single sigmoid neuron for output.

The small size of this network allows it to be trained on a small number of hand-labeled spots. We manually classified 1250 voxels from 4 time points, resulting in a training dataset with 227 spots and 1023 “not spots”. We trained the model by minimizing the binary cross-entropy loss using the Adam optimizer with a learning rate of 10^−4^ and batch size of 8 in 5-fold cross validation. We augmented the training data with random rotations. To offset class imbalance during training, we used class weights equal to half the inverse fraction of each class in the training data. After 100 training epochs, the validation loss decayed to 0.21 ± 0.05 (mean ± standard deviation) and remained stable (Supplemental Fig 3C). The final average validation area under the curve (AUC) was 0.97 ± 0.01.

During spot analysis, the initial set of detected candidate spots is filtered down using a probability threshold of 0.7.

#### Quantification:

Spot quantification occurs through particle localization, background estimation and subtraction, and then integration over a defined ellipsoid of size (4×4×4) pixels^3^, or (2×2×4) microns^3^. Our pipeline contains two particle localization algorithms: least-squares Gaussian fitting and the radial center algorithm (Parthasarathy 2012), which is an analytic estimate of particle center based on radial symmetry. Both methods are highly accurate. The radial center algorithm is inherently much faster, as it requires no fitting. However, we implemented multi-processor parallelization of *scipy’s least_squares* function that is quite fast in practice. As discussed more below, Gaussian fitting has the benefit that the best-fit width parameters have strong predictive power in further discriminating between true and false spots. Throughout this paper, we used the Gaussian fitting method.

Importantly, because of the background MCP-mNeonGreen expression in and around each nucleus has spatial structure, straightforward application of both particle localization algorithms, which assume uniform background with Poisson noise, fail on the majority of spots. To minimize the structured background, we first perform Difference of Gaussians filtering on the spot voxel and then estimate particle centers.

Next, we estimate the background levels for each spot individually and subtract that background from the spot pixel intensities. Background estimation can be done in one of two ways. The first way is to re-fit a 3D Gaussian to the spot in real space, but with fixed center and width parameters, extracting just an offset and amplitude. Gaussian fitting is the standard way to estimate background levels of MS2 spots (Garcia et al. 2013). The second way is to take a “shell” of pixels of a fixed width and distance from the spot center and average the intensity of the shell. Using a shell of inner width = 4 pixels and outer width = 6 pixels, we find that the two methods of background estimation strongly agree with one another (Supplementary Fig 3F, R^2^ = 0.94). In all data in this paper, we used the shell method, as it is faster.

With the background estimate in hand, we subtract this background from all pixels in the voxel, enforcing non-negativity. We then define an ellipsoid of size 4×4×4 pixels^3^ and sum the background-subtracted intensities.

To assess the accuracy of this quantification procedure, we generated a library of synthetic spots with known integrated intensity and realistic background and noise (Supplemental Fig. 4A). Specifically, we modeled spots using a Gaussian point spread function (PSF) and a constant offset. We then modeled the structured background by adding to this Gaussian an exponential function with decay length equal to 10 pixels and a random orientation in 3D. Finally, we modeled shot noise by assigning to each pixel value a Poisson random number with mean equal to the intensity of the noise-less pixel.

We ran our spot quantification pipeline on this library and assessed intensity accuracy as a function of spot amplitude (which sets the signal-to-noise ratio) and the amplitude of the exponential gradient. We measured the relative accuracy of the intensity measurement, defined as (measured intensity - true intensity) / true intensity, across spot parameters and also using two different spot localization methods: first, Gaussian fitting on the raw pixels, and second, Gaussian fitting on Difference-of-Gaussians (DoG)-filtered images, the latter to remove the structured background. We found that localization using DoG-filtered images led to considerably enhanced accuracy across spot parameters (Supplemental Fig. 4B).

To make contact with the experimental data, we defined two readily measured parameters: signal:background ratio (where signal is the mean background-subtracted spot pixel intensity) and the “structuredness” of the background, defined as the ratio of the variance:mean of the shell pixels that define the background. For a uniform background with Poisson noise, the structuredness is 1. Binning our simulations in this 2D space, we created an accuracy regime diagram (Supplemental Fig. 4C). Averaging across all spots in our dataset, we found that the experimental data had a signal:background value of 0.35 ± 0.24 (mean ± standard deviation), i.e., 35% above background, and a structuredness value of 40 ± 21, reflecting the highly structured MCP background visible in the spot images. Nevertheless, with DoG-based localization and our Gaussian combined with our exponential model of spots, we infer that our spot intensity quantification is highly accurate, with an relative error of at most 40%, but typically around 5%.

### Trace assembly

MS2 traces are assembled by assigning spots to nuclei and linking them through time using the nuclear tracking information. First, we check if a spot’s centroid falls within a region identified as a nucleus by the “Segments” label matrix that is output from *dexp-dl*. If a spot does not fall within a nucleus, then we perform a search for the nearest nucleus within a cube of size 11 pixels in x and y and 7 pixels in z, centered on the spot. If multiple spots are assigned to the same nucleus, we pick the one with the highest probability value from the neural network classifier.

After assigning each spot to a nucleus, we assemble a draft of the traces and begin iteratively refining them. In each iteration we perform the following steps. We identify regions of transcriptional activity by thresholding a moving average of the trace using a window of 5 time points and a threshold of 1 a.u. (effectively any non-zero point passes the threshold). For each point in the “on” region of the trace such that the raw trace has a value of zero and has an adjacent point with a value greater than zero, we attempt to fill in that point. We extract a voxel centered on the location of the adjacent point but at the “empty” time point. We then run this voxel through our spot classification and quantification routines. We accept or reject the spot using either the spot classifier or quantification parameters. For all data in this paper, we kept only spots for which all Gaussian width parameters (sigma_x, sigma_y, and sigma_z) fell within the range 0.5 and 3.0 pixels, which we found to be an effective filter by manual inspection.

Upon iterating this process, the set of found points that pass the given criteria will converge. For all data in this paper, we used 10 iterations.

After the traces are assembled, we can make further cuts by rejecting spots based on spatial location. Previous measurements of protein levels (Delaune et al. 2012) and smFISH counts (Keskin et al. 2018) indicate that *her1* transcription halts after somite boundary formation. For reasons unknown, we found that background levels of MCP-mNeonGreen increase in somites after boundary formation, leading to increased false positive detections in this region. We therefore reject spots that are greater than 40 microns anterior of the last formed somite, as measured along our defined anterior-posterior axis (see below). Similarly, we restrict our analysis to spots detected within the PSM and tailbud tissues by rejecting spots that lie greater than 50 microns from the anterior-posterior axis. Finally, for analysis of single-cell traces, we keep only traces with greater than 10 spots.

### Spot intensity uncertainty

The uncertainty in individual spot intensity was estimated using a method based on (Garcia et al. 2013). A full derivation of the uncertainty is given in the Appendix. In brief, the uncertainty was assumed to be dominated by the error in background estimation. This error is measured by considering the time evolution of the background around each spot, fitting a mean trend using a 4th order polynomial, and computing the RMS deviation of background levels from this trend. In contrast to the original method (Garcia et al. 2013), we use a 4th order polynomial as opposed to a spline as we found that additional degrees of freedom in the trend model led to worse performance in a cross-validation scheme (Appendix).

In addition, and in contrast to previous works, due to the increased difficulty of the spot detection problem we further incorporate uncertainty in spot detection via the empirically measured false-positive and false-negative rates (Appendix). Combining both of these sources of uncertainty, we arrive at the final uncertainty for an MS2 traces as

(1) $$\sigma =\sqrt{{\left(\sigma_{l}\right)}^{2}\left(1-p\right)+I^{2}p\left(1-p\right)},\;\;\;I>0\;\sigma =\bar{I}\sqrt{n\left(1-n\right)},\;\;\;\;\;\;\;I=0$$

where $\sigma_{I}$ is the uncertainty from background fluctuations, p is the false-positive rate of detection, n is the false-negative rate of detection, I is the intensity of the individual spot, and $\bar{I}$ is the mean intensity of the trace. From analysis of manually-curated traces, we measured p=0.04 and n=0.08. $\sigma_{I}$ depends on the trace, but is typically 1–10% of $\bar{I}$. See Appendix for more details.

### Anterior-posterior axis

We define an anterior-posterior axis using a combination of manually placed points and spline interpolation. Using the interactive data viewer *napari* (Sofroniew et al. 2022), we construct a coarse anterior-posterior axis by manually placing around 10 points that span from the last formed somite (which becomes the origin) to the tip of the tail bud in the first time point of the timeseries. We place these points in 3D using a *napari Points layer*, viewing one slice at a time, roughly following the notochord. Keeping track of the location of that last formed somite, we proceed to define coarse anterior-posterior axes every 50 time points until the end of the movie. We then refine these anterior-posterior axes through spline interpolation. First, we interpolate the anterior-posterior axis of each time point, going from 10 to 100 spatial positions, using a B spline of degree 2. Then, for each 100 points along the new anterior-posterior axis, we interpolate over time, using a B spline of degree 1. We use *scipy’s splprep* function to define the spline.

### Protein signal prediction

We use a linear model of transcription, translation, and protein maturation to predict what mRNA (*m*) and protein signal (*p*) would be created by our MS2 traces (*x*), assuming that the fluorescent intensity of the MS2 signal is proportional to the instantaneous rate of transcription (Lammers, Galstyan, et al. 2020). Specifically, we model the system according to

(2) $$\frac{dm}{dt}=r_{m}x-\gamma_{m}m$$

(3) $$\frac{dp}{dt}=r_{p}m-\gamma_{p}p$$

The key parameters are the decay rates (*γ*_*m*_, *γ*_*p*_) of her1 mRNA and protein, respectively, which we take from reference (Lewis 2003) to both be 0.23 1/min. The production rates (*r*_*m*_, *r*_*p*_) simply set the concentration scales of mRNA and protein, respectively, which here can be taken as arbitrary. The initial conditions of total mRNA and protein are unknown parameters. In the Appendix, we show that the predicted mRNA and protein signals become independent of the initial condition on the time scale of the decay constants. Therefore, for simplicity, we pick initial conditions of mRNA(t=0)=protein(t=0)=0 and ignore approximately the first cycle of predicted signals.

In Fig. 1B, we use this model to demonstrate the low-pass filtering effect of the central dogma (more details in Appendix). We generated toy example MS2 traces consisting of either a sine wave or a square wave and used these as inputs to the protein prediction model, which outputs smooth oscillations in both cases.

### Burst calling and calculating number of bursts per oscillation

We implemented a conservative burst calling algorithm based on thresholding a moving average of the *her1*-MS2 traces. The moving average was computed via convolution with a uniform kernel of size 3 time points, and we used a threshold of 1 a.u., effectively grouping any non-zero signal into a burst. With this algorithm, fluctuations in *her1* transcription are determined as corresponding to distinct bursts only if they are separated by 3 time points of inactivity.

We defined oscillations using the predicted protein signal. We used *scipy’s find_peaks* function to identify peaks in the predicted protein oscillation. We then collected burst start and end times and assigned the bursts starting between the previous protein peak and the current protein peak to the current protein peak. With all bursts being assigned to a protein peak, we then computed a histogram of the number of bursts per protein peak, which we call the number of bursts per oscillation.

### Generalized bursting model with feedback

Details of the mathematical model and its implementation are found in the Appendix, Section 3. In brief, we extended the canonical two-state bursting model to allow for protein concentration to increase the rate of transcription, decrease the rate of OFF→ ON events, or increase the rate of ON → OFF events according to Hill functions.

#### Model parameters:

Model parameters were chosen manually to produce simulated MS2 traces that qualitatively resemble the experimental data and exhibit statistically increased regularity in the resulting oscillations compared to unregulated bursting. To quantify the degree of regularity in the resulting oscillations, we computed the coefficient of variation (CV, standard deviation/mean) of the protein oscillation periods (see below for details). Lower values of the period CV indicate more regular oscillations. As a lower bound on the period CV to be expected for unregulated bursting, we use the minimum value of the CV of promoter state periods, which in the unregulated bursting model is approximately 0.7. Thus, oscillations with a period CV of less than 0.7 are more regular than can be accounted for by unregulated bursting. For all simulations, the mRNA and protein decay rates were both set to 0.23 1/min (Lewis 2003). For the simulations in Figure 6, the following parameters were used: (B) Amplitude regulation: the transcription rate is repressed by the protein according to a Hill function, *f*(*p*) = 1/(1 + (*p*/*K*_*D*_)^*n*^), evaluated at a delayed protein value p(t-*τ*). Parameters: *k*^*+*^=1.6 1/min, *k*^−^=3.2 1/min, *r*_*0*_=400 a.u./min, *K*_*D*_=1.0, *n*=2, *τ*=8 min. Protein period CV=0.5. (C) Frequency regulation: *k*_*on*_ is repressed according to the same Hill function *f*(*p*). Parameters: *k*^*+*^=300 1/min, *k*^−^=0.08 1/min, *r*_*0*_=1.0 a.u./min, *K*_*D*_=0.01, *n*=2. Protein period CV=0.3. (D) Duration regulation: *k*_*off*_ increases with protein level according to an increasing Hill function *g*(*p*) = (*p*/*K*_*D*_)^*n*^/(1 + (*p*/*K*_*D*_)^*n*^). Parameters: *k*^*+*^=0.08 1/min, *k*^−^=0.2 1/min, *r*_*0*_=1.0 a.u./min, *K*_*D*_=12.0, *n*=12. Protein period CV=0.5.

#### Simulated MS2 signals:

From each simulation we extracted a time series of the promoter state variable, which fluctuates between 0 (“OFF”) and 1 (“ON”). Using these promoter state traces, we simulated realistic MS2 traces using the model of (Lammers, Galstyan, et al. 2020) that encapsulates the effect of RNA polymerases being loaded on and off the gene with a memory kernel of normalized length *w*. We further included multiplicative Gaussian noise with standard deviation σ_*MS2*_ to simulate fluorophore emission and measurement uncertainty, and a detection threshold, below which the simulated MS2 signal was set to zero. We used the following parameters: *w* = 3.65, σ_*MS2*_ = 0.2, and detection threshold = 0.2 * *w* * max(*r*_*m*_), where *r*_*m*_ is the mRNA production rate in equation 37 of the Appendix and is time-dependent in the case of amplitude regulation.

#### Protein period regularity measurement:

To assess the regularity of simulated protein oscillations in our model, we used the *scipy* find_peaks function with prominence = 0.01 to identify the locations of peaks in protein oscillations. We then defined the protein period as the intervals between peaks and computed the coefficient of variation (CV, standard deviation/mean) of these periods.

#### Unregulated two-state bursting model predictions:

We compared simulated and experimentally measured burst interval distributions in the unregulated two-state model of transcriptional bursting. In this model, the gene can be in one of two states, ON or OFF. The gene stochastically switches between these states according to a simple Markov process with rates *k*_*on*_ and *k*_*off*_. Thus, the time spent in each state is exponentially distributed with mean equal to the inverse rate. We empirically estimated *k*_*on*_ and *k*_*off*_ as the inverse of mean of the inactive and active durations, respectively.

## Supplementary Material

Supplement 1

Supplement 2

Supplement 3

Supplement 4

Supplement 5

Supplement 6

Supplement 7

Supplement 8

Supplement 9
