# Supplementary material for: Single-cell transcriptional dynamics in a living vertebrate
Source: bioRxiv. 2025 Jun 11:2024.01.03.574108. Originally published 2024 Jan 4. Preprint. [Version 2] doi: 10.1101/2024.01.03.574108 (PMC10802376; doi:10.1101/2024.01.03.574108)
Supplement: Supplement 10 [file NIHPP2024.01.03.574108v2-supplement-10.pdf]

bioRxiv preprint doi: https://doi.org/10.1101/2024.01.03.574108; this version posted June 11, 2025. The copyright holder for this preprint (which was not certified by peer review) is the author/funder, who has granted bioRxiv a license to display the preprint in perpetuity. It is made available under aCC-BY 4.0 International license.

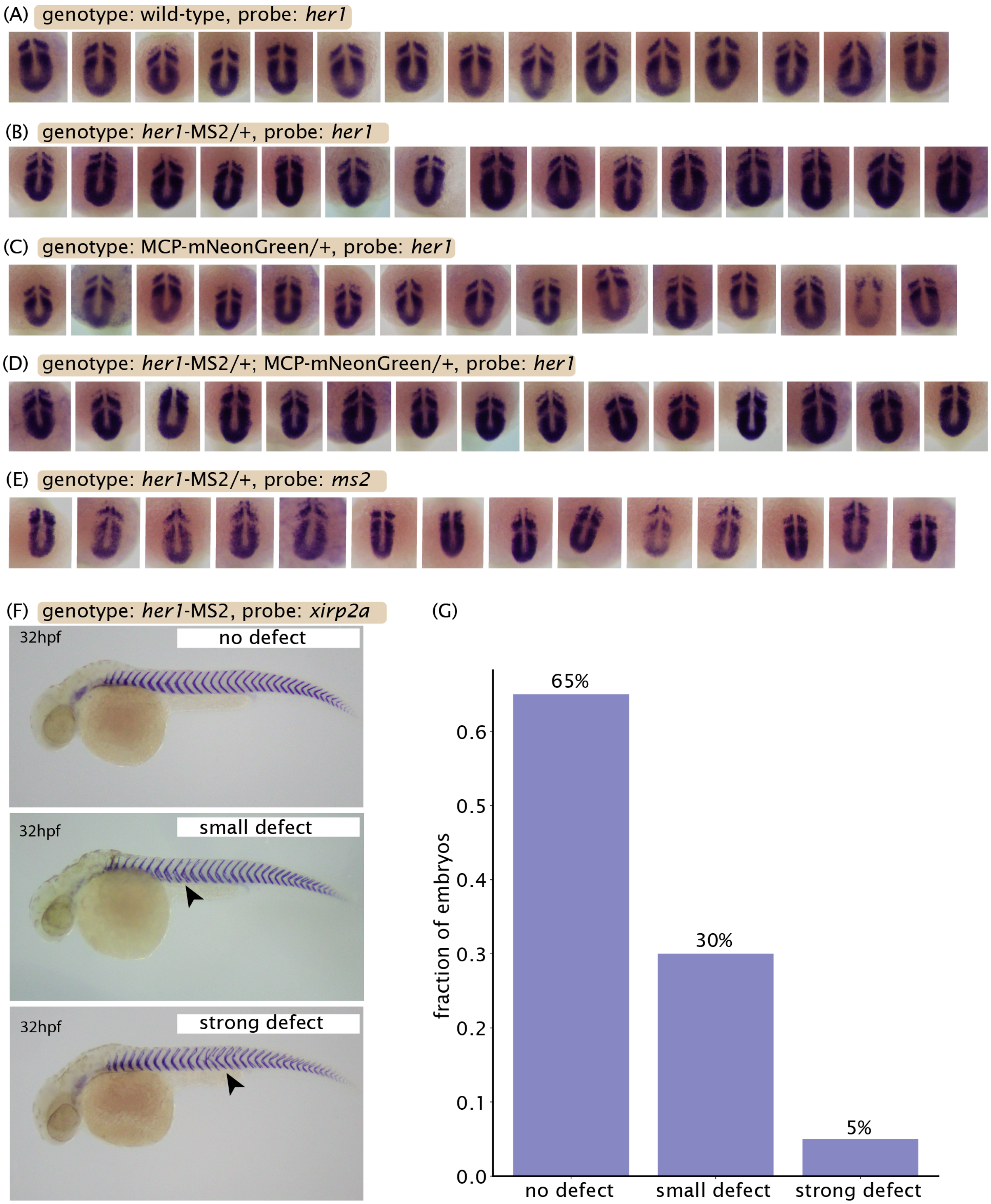

Supplemental Figure 1: Controls show overall healthy somitogenesis of *her1*-MS2 and MCP-mNeonGreen transgenic lines. (A)-(D): In situ hybridization assays against *her1* and MS2 in various genotypes at the 12-14 somite stage embryos showing qualitative agreement of

bioRxiv preprint doi: https://doi.org/10.1101/2024.01.03.574108; this version posted June 11, 2025. The copyright holder for this preprint (which was not certified by peer review) is the author/funder, who has granted bioRxiv a license to display the preprint in perpetuity. It is made available under aCC-BY 4.0 International license.

the *her1* expression pattern in both wild-type and transgenic fish. All embryos are offspring of a cross of *her1*-MS2 and MCP-mNeonGreen heterozygous animals. (A) “Wild-type” embryos lacking both markers for MS2 and MCP show normal *her1* expression patterns (Schröter et al. 2012). (B) “*her1*-MS2” embryos carrying only the MS2 transgene show healthy *her1* expression. (C) “MCP-mNeonGreen” embryos carrying only the MCP transgene show healthy *her1* expression. (D) Embryos carrying both transgenes show healthy *her1* expression. (E) In situ hybridization assay against the MS2 sequence in *her1*-MS2 embryos showing that the reporter qualitatively recapitulates the endogenous *her1* expression pattern. (F) *In situ* hybridization assay for the somite boundary marker *xirp2a* (Riedel-Kruse, Müller, and Oates 2007), showing examples of no defect, small defect, strong defect. When defects do occur, they are limited to the trunk region, which completes somite formation before our imaging experiments begin and is thus not included in our analysis. (G) The majority of animals show no defects at 32 hours post fertilization.

bioRxiv preprint doi: https://doi.org/10.1101/2024.01.03.574108; this version posted June 11, 2025. The copyright holder for this preprint (which was not certified by peer review) is the author/funder, who has granted bioRxiv a license to display the preprint in perpetuity. It is made available under aCC-BY 4.0 International license.

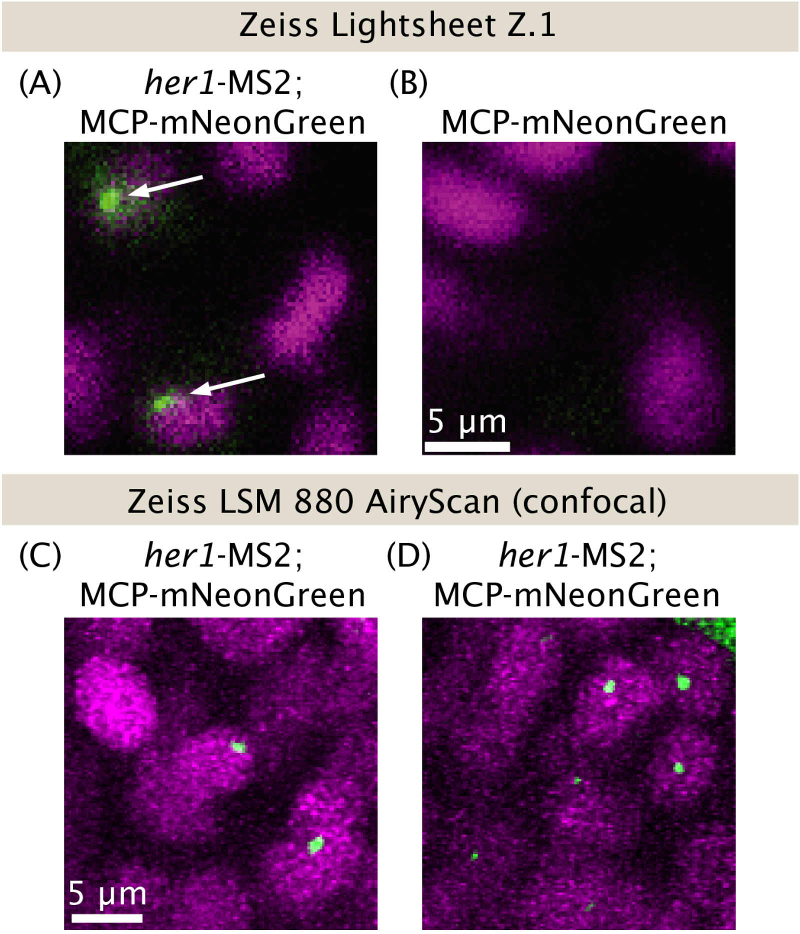

Supplemental Figure 2: Characterization of spots on commercial light sheet and confocal fluorescence microscopes. (A,B) Spots (white arrows) are detected on the Zeiss Z.1 Lightsheet but only when the reporter gene is present. MCP-mNeonGreen fish show no puncta in the absence of the *her1*-MS2 reporter. Single z-slices of embryos with MCP-mNeonGreen (green) and h2B-mScarlet (magenta) as a nuclear marker (A) with *her1*-MS2 or (B) without *her1*-MS2. Contrast was adjusted manually such that background MCP levels were approximately equal in both images. (C)-(D) Two examples of spots on a Zeiss LSM 880 confocal microscope with AiryScan processing.

bioRxiv preprint doi: https://doi.org/10.1101/2024.01.03.574108; this version posted June 11, 2025. The copyright holder for this preprint (which was not certified by peer review) is the author/funder, who has granted bioRxiv a license to display the preprint in perpetuity. It is made available under aCC-BY 4.0 International license.

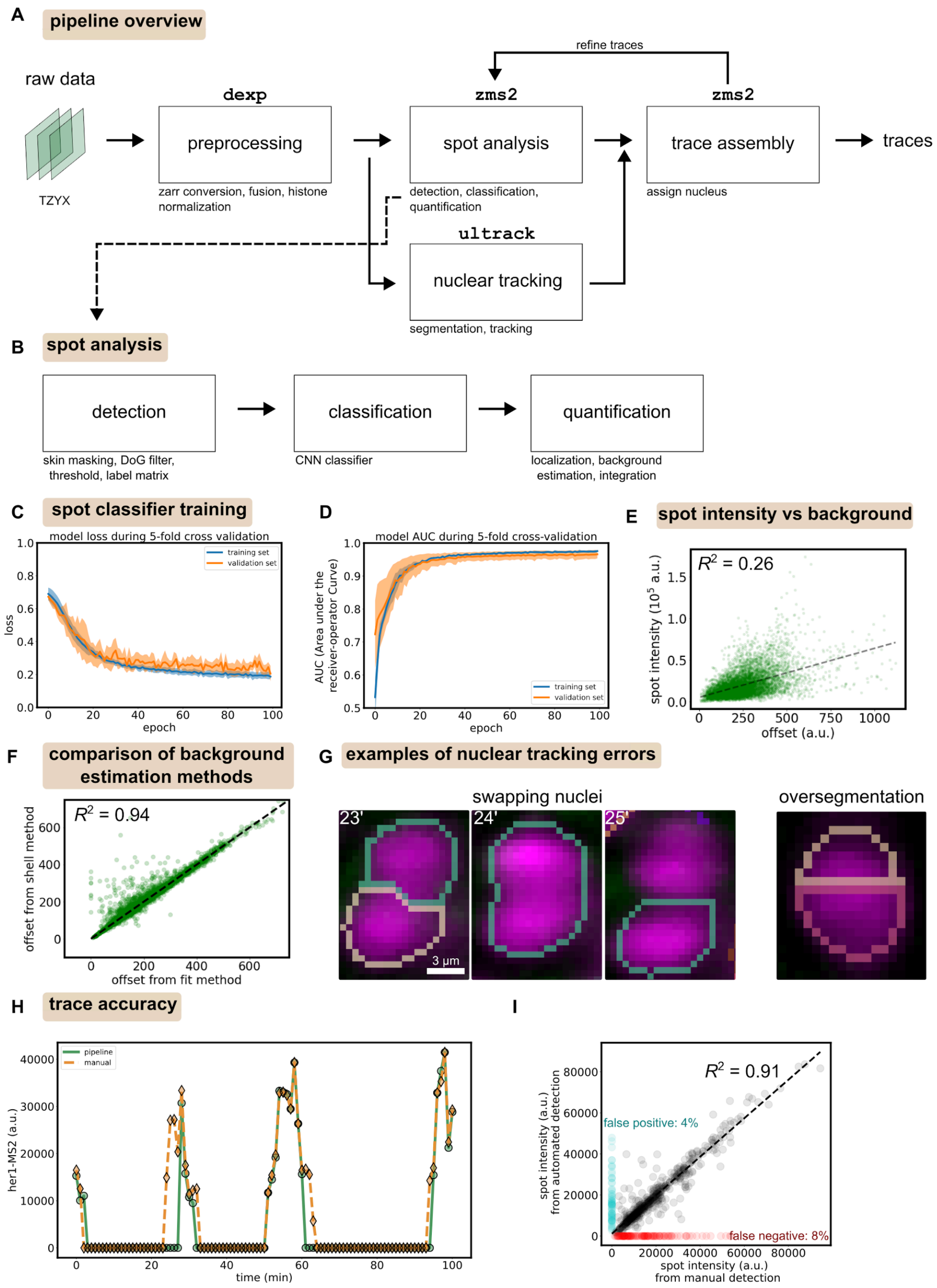

bioRxiv preprint doi: https://doi.org/10.1101/2024.01.03.574108; this version posted June 11, 2025. The copyright holder for this preprint (which was not certified by peer review) is the author/funder, who has granted bioRxiv a license to display the preprint in perpetuity. It is made available under aCC-BY 4.0 International license.

Supplemental Figure 3: Overview of image analysis pipeline and its accuracy. (A) Schematic of the full pipeline. The pipeline takes two 4D image stacks as input, one for the nuclear marker and one for the MS2-MCP reporter per light sheet view. Preprocessing using the *dexp* package (Yang et al. 2022) involves conversion to the *zarr* file format, fusion of multiple light sheet views (if necessary), and normalization of the nuclear marker channel to prepare for nuclear segmentation. After preprocessing, the nuclear and spot channels are split, with the nuclear channel undergoing segmentation and tracking via *ultrack* (Jordao Bragantini et al. 2024) and the spot channel passing through our spot pipeline, called *zms2*, which detects and analyzes MS2 spots. The outputs of these packages are combined into traces of spot fluorescence for a given nucleus as a function of time. Traces are then refined using an iterative computational algorithm. In brief, the algorithm identifies abrupt gaps in traces and attempts to fill them in by extracting a voxel around the expected spot location, passing it back through the classification and quantification modules, and seeing if the spot passes a set of criteria (see the "Spot analysis" section of the Methods). (B) Schematic spot analysis section of the full pipeline. Candidate spots are detected and then classified as a true spot or a false-positive detection using a binary Convolutional Neural Network (CNN) classifier. The culled list of spots then undergo quantification, where the total fluorescence intensity of the signal is computed via spot localization, background estimation and subtraction, and then summing pixel values within a defined ellipsoid (see the "Spot analysis" section of the Methods). DoG=Difference of Gaussians, CNN=Convolutional Neural Network. Skin masking=computational removal of bright and irrelevant skin cells. Thresholding occurs on the DoG-filtered image to create a binary mask, which is transformed to a label matrix that assigns a unique identifier to each candidate spot. (C) Binary cross-entropy loss function of convolutional neural network spot classifier over training shows that the model converged with minimal overfitting. Solid lines are means and shaded error bars standard deviations over 5 folds of cross validation. Blue=training set ("train"), Orange=validation set ("val"). CV=cross validation. (D) Area Under the receiver-operator Curve (AUC), a metric of model performance that balances false positive and false negative detections, during training ("epoch" = time in the training process). The validation AUC converged to 0.97 ± 0.01, indicating very high classification performance (a perfect classifier has AUC=1, an unbiased random guess has AUC=0.5). (E) Spot intensity vs. background level ("offset") showing an extremely weak correlation (R^2=0.26), which is indicative of MCP saturation and reliable measures of absolute fluorescence intensity. (F) Comparison of two methods for background estimation, described in the Methods section. "Shell" refers to taking the mean of pixel values in a shell around the spot. "Fit" refers to fitting a Gaussian with an offset. We find that the two methods give very similar results, with the data clustering closely around the 1:1 line (black dashed line) and an R^2 of 0.94. The best fit slope is 1.049 +/- 0.003. (G) Examples of nuclear tracking errors, including when the algorithm swaps the identities of nearby nuclei (left), and when it splits a single nucleus into two segments (right). Shown are single z-slices of histones (magenta), with the algorithm-assigned identity of each nucleus marked by a unique colored outline. MCP-mNeonGreen background is faintly visible in green. (H) Example comparison of a pipeline-derived trace (green solid line) and the corresponding manually-derived trace (orange dashed line), obtained by manually clicking on spots in images and passing these spots through the quantification pipeline. (I) Comparison of spot intensities

bioRxiv preprint doi: https://doi.org/10.1101/2024.01.03.574108; this version posted June 11, 2025. The copyright holder for this preprint (which was not certified by peer review) is the author/funder, who has granted bioRxiv a license to display the preprint in perpetuity. It is made available under aCC-BY 4.0 International license.

between pipeline and manual traces across 728 spots from 26 nuclei. Cases where both methods detect a spot are colored in black. Linear regression to these points shows strong correlation (R^2 = 0.91). Cases in which a human detected a spot and the pipeline did not (false negative) are colored in red. These cases are 8% of all spots. Cases in which a human did not detect a spot but the pipeline did (false positive) are colored in cyan. These cases are 4% of all spots.

bioRxiv preprint doi: https://doi.org/10.1101/2024.01.03.574108; this version posted June 11, 2025. The copyright holder for this preprint (which was not certified by peer review) is the author/funder, who has granted bioRxiv a license to display the preprint in perpetuity. It is made available under aCC-BY 4.0 International license.

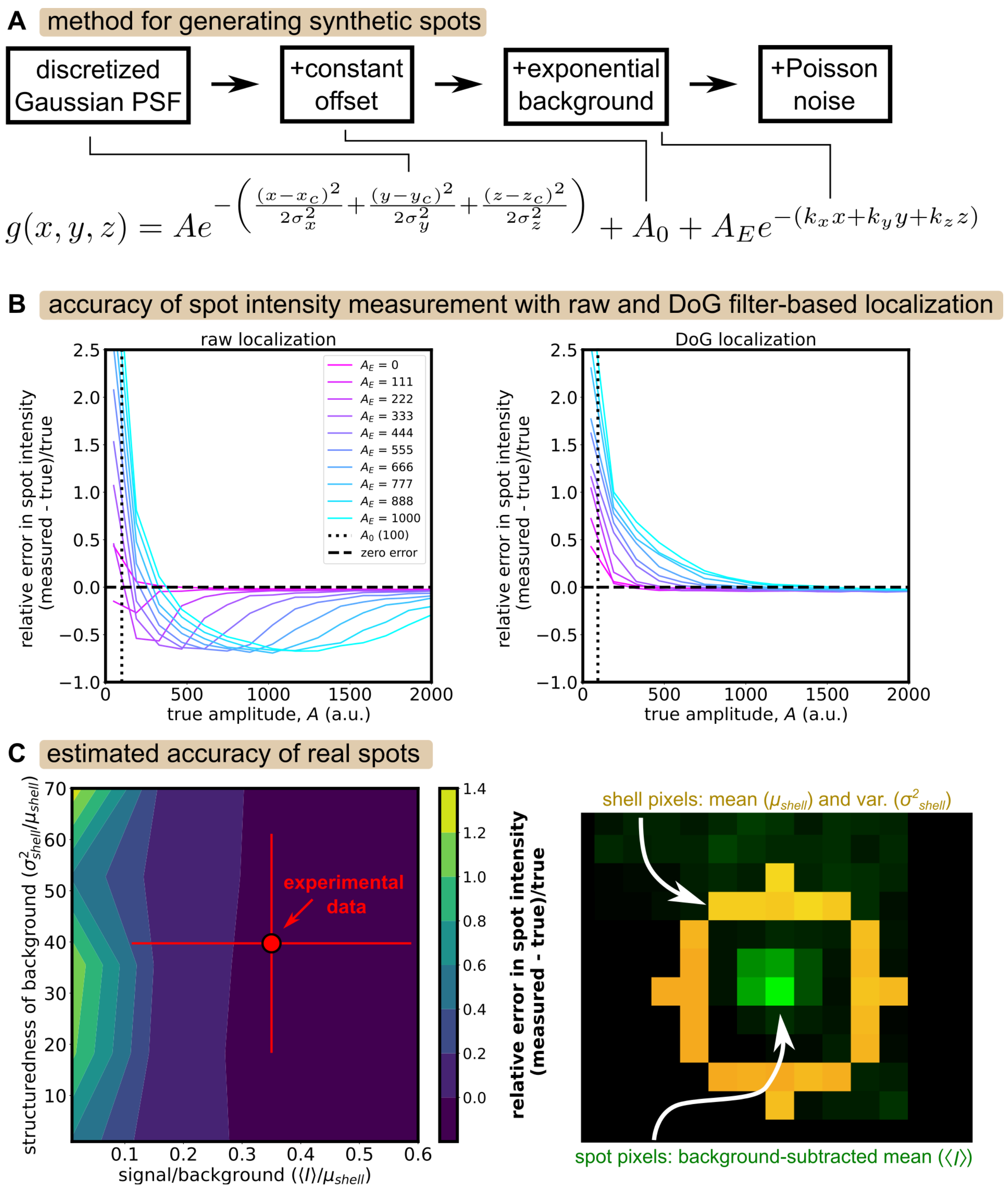

Supplemental Figure 4: Characterization of spot intensity measurement accuracy using synthetic spots. (A) Schematic of method for generating synthetic spots with structured background. The Point Spread Function (PSF) is modeled as a Gaussian function, to which we add a constant offset and an exponentially decaying background, the latter being a simple model of the structured MCP background we observe in real spots (not shown). The

bioRxiv preprint doi: https://doi.org/10.1101/2024.01.03.574108; this version posted June 11, 2025. The copyright holder for this preprint (which was not certified by peer review) is the author/funder, who has granted bioRxiv a license to display the preprint in perpetuity. It is made available under aCC-BY 4.0 International license.

wavevector, *k*, of the background is taken to have a length of 10 pixels (roughly the length of the spot in voxels) and a random direction. The pixels of the final image are then drawn from a Poisson distribution with mean equal to *g(x,y,z)*. (B) Relative error in spot intensity (measured - true)/true vs true spot amplitude (the parameter *A* in the equation in panel (A)) for different strengths of the exponential background (via the parameter A_E). Left: spot localization via Gaussian fitting of the raw image. Right: spot localization via Gaussian fitting of a Difference-of-Gaussians (DoG)-filtered image. After localization, background estimation is done with the “shell” method described in the Methods. Intensity is then the sum of background-subtracted pixels inside a defined ellipsoid. Intensity errors are larger when localization is done on the raw pixels, due to the structured background. (C) Left: Placing the experimental data (red circle=mean, red bars=standard deviation across all spots) in a regime diagram describing relative accuracy of the intensity measurement (heatmap) as a function of signal/background and “structuredness” of the background. The x-axis, signal/background, is the average background-subtracted value of spot pixel intensities, e.g., 0.1 = 10% above background. The y-axis, structuredness of background, is the variance of shell pixel intensity divided by the mean of the shell pixel intensity . For a uniform background with Poisson noise, the structuredness will be 1. The experimental data has a background with structuredness of approximately 40 on average. The simulations from (B) were binned into a 2D histogram according to their signal/background and structuredness levels to create the final heatmap of relative error in measured spot intensity, defined as (measured intensity - true intensity)/(true intensity). For example, a relative error of 1.0 means the algorithm overestimated the total spot intensity by 100%, or a factor of 2. This heatmap illustrates that, based on our model of spots, the DoG-based localization method should lead to highly accurate intensity estimations, with a relative error of less than 10%. Right: Schematic of the parameters used to define the axes of the heatmap on the left, using a single z slice of a real spot (green) with shell pixels highlighted in orange.

bioRxiv preprint doi: https://doi.org/10.1101/2024.01.03.574108; this version posted June 11, 2025. The copyright holder for this preprint (which was not certified by peer review) is the author/funder, who has granted bioRxiv a license to display the preprint in perpetuity. It is made available under aCC-BY 4.0 International license.

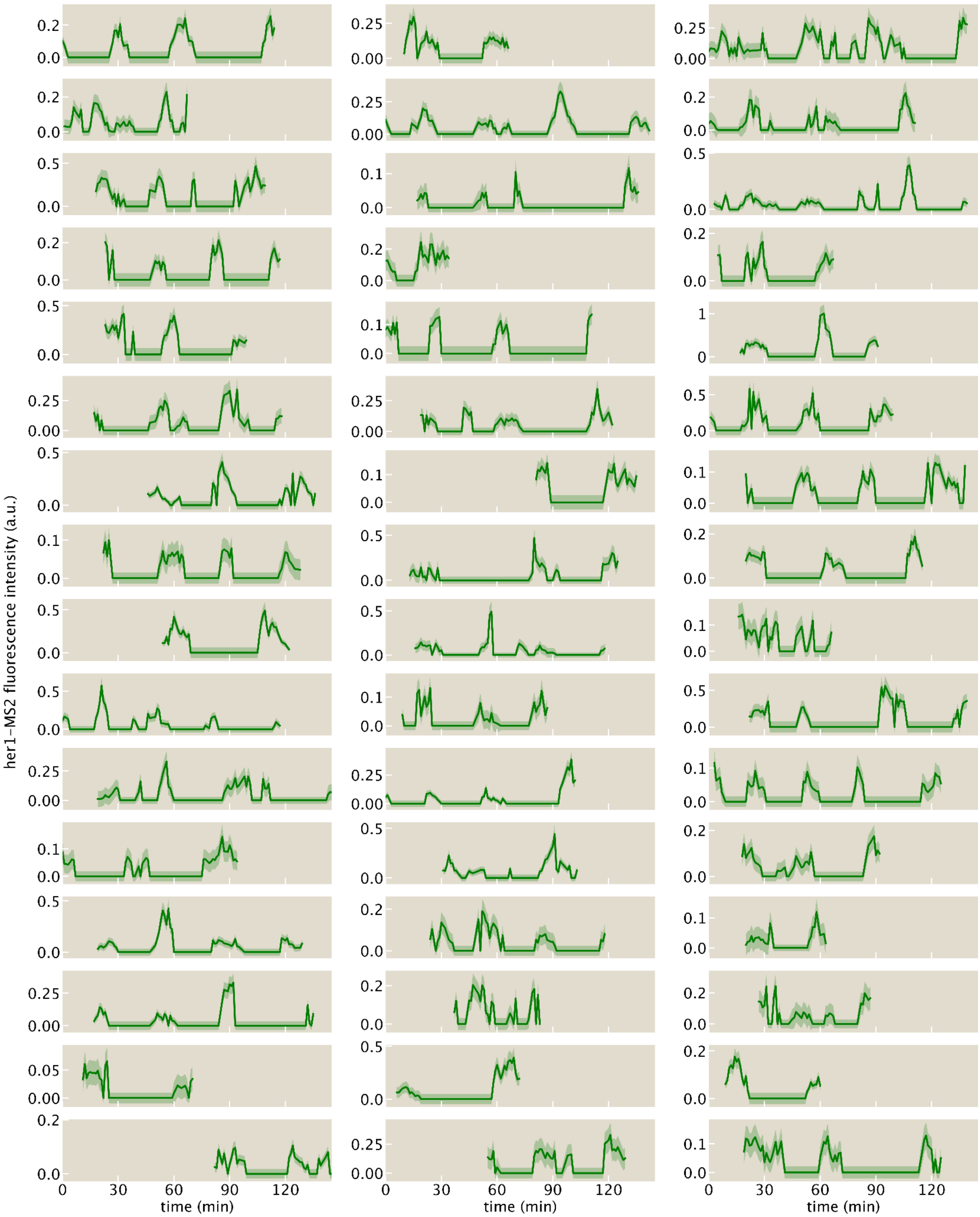

Supplemental Figure 5. 48 randomly chosen traces with at least 20 spots per trace.

bioRxiv preprint doi: https://doi.org/10.1101/2024.01.03.574108; this version posted June 11, 2025. The copyright holder for this preprint (which was not certified by peer review) is the author/funder, who has granted bioRxiv a license to display the preprint in perpetuity. It is made available under aCC-BY 4.0 International license.

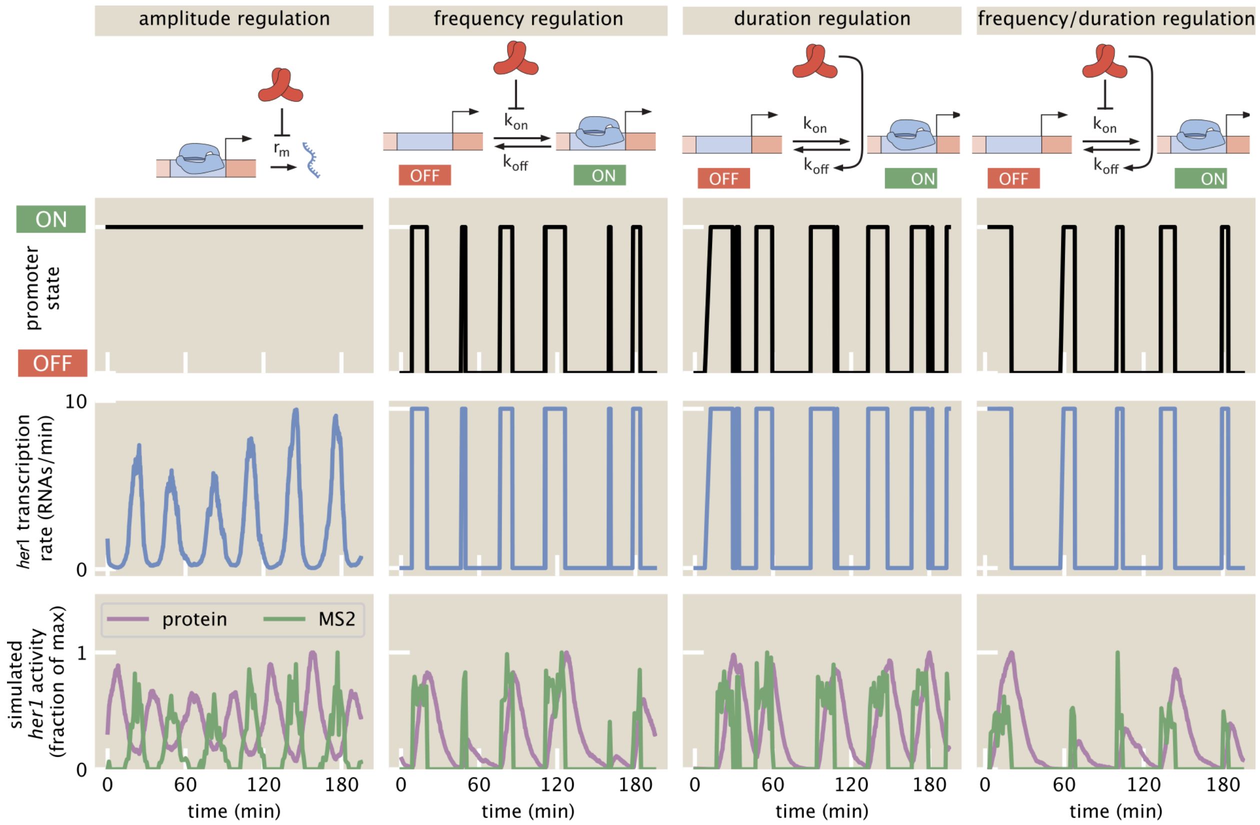

Supplemental Figure 6. Examples of simulated traces for different models. Each column corresponds to a different model of autorepression (see Fig. 6). Top row: dynamics of promoter state switching. Middle row: instantaneous transcription rate. Bottom row: simulated traces of the expected *her1*-MS2 signal (Methods) and Her1 protein concentration, normalized to maximum. Parameters are the same as in Fig. 6, described in the Methods section.

bioRxiv preprint doi: https://doi.org/10.1101/2024.01.03.574108; this version posted June 11, 2025. The copyright holder for this preprint (which was not certified by peer review) is the author/funder, who has granted bioRxiv a license to display the preprint in perpetuity. It is made available under aCC-BY 4.0 International license.

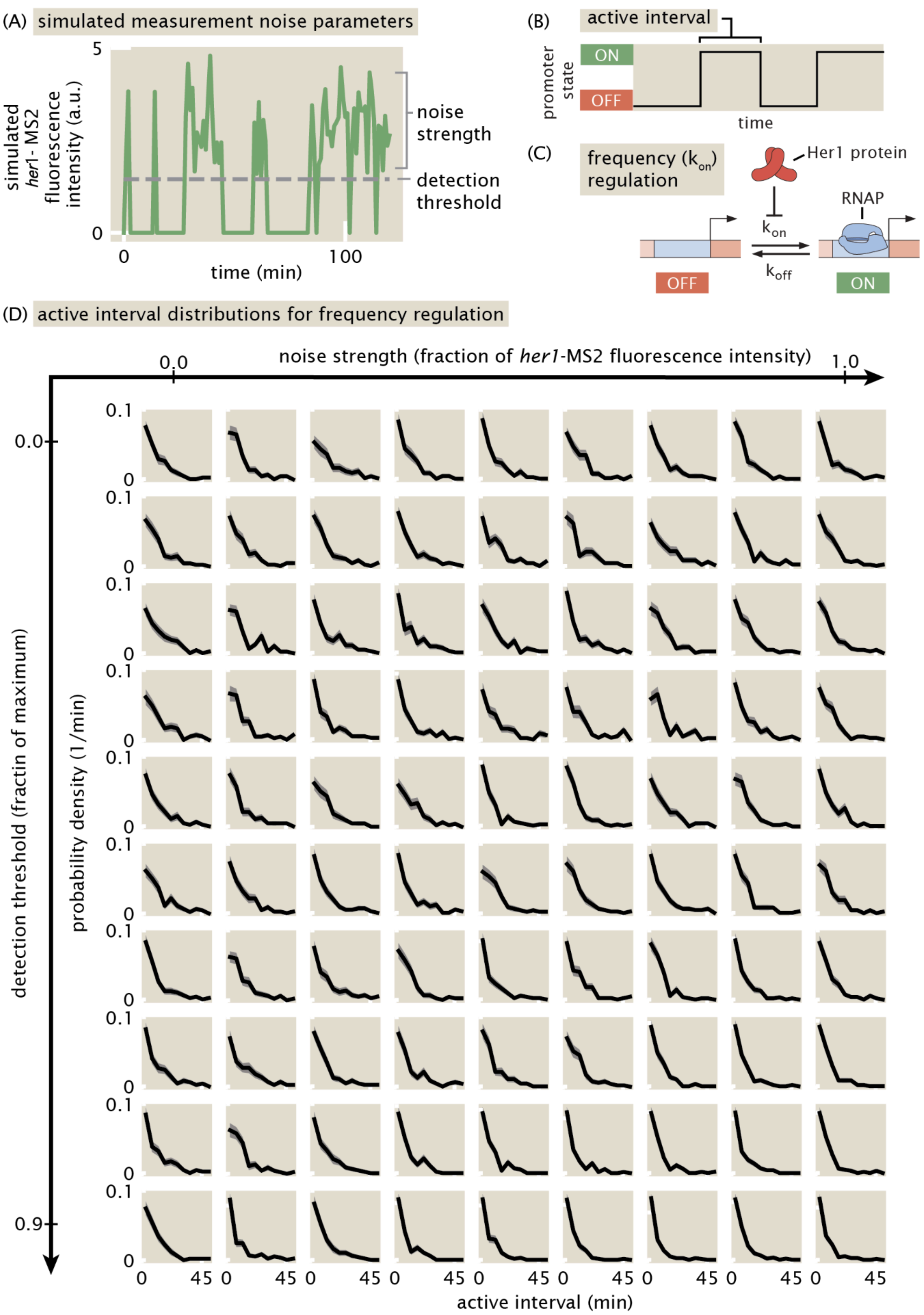

bioRxiv preprint doi: https://doi.org/10.1101/2024.01.03.574108; this version posted June 11, 2025. The copyright holder for this preprint (which was not certified by peer review) is the author/funder, who has granted bioRxiv a license to display the preprint in perpetuity. It is made available under aCC-BY 4.0 International license.

Supplemental Figure 7: Active interval distributions for the frequency regulation bursting model remain exponential for a wide range of simulated measurement noise parameters. Our goal is to assess the impact of the MS2 measurement process on our ability to correctly identify the shape of burst interval distributions. (A) Simulated *her1*-MS2 trace illustrating the two measurement noise parameters: noise strength, which determines the level of measurement noise within a burst, and detection threshold, which determines how strong the *her1*-MS2 signal must be to be detected. Specifically, "noise strength" is the amplitude of multiplicative Gaussian noise added to the simulated *her1*-MS2 trace; we vary the noise strength from 0.0 to 1.0. The "detection threshold" is implemented by setting all values less than the detection threshold to zero; we vary the detection threshold from 0.0 to 0.9 times the maximum *her1*-MS2 signal. Other model parameters are described in the "Generalized bursting model with feedback" section of the Methods. These parameter values were chosen to produce quasi-regular oscillations while being consistent with the literature where possible. (B) In this figure, we focus on the analysis of the active burst interval (also known as burst duration), defined as the duration of time the promoter is in the ON state. (C) We also focus here on the case of frequency regulation, where Her1 proteins regulate the rate of the promoter turning on, k_{on}. In the absence of measurement noise, the active interval distribution for frequency regulation is exponential, since the active interval is set by k_{off}, not k_{on}, and here k_{off} is constant. (D) We explored how measurement artifacts affect the shape of this distribution by creating a 2D grid of active interval distributions, with increasing noise strength to the right and increasing detection threshold going down. The active interval distribution remains exponential over all of the explored parameter space, indicating that the degree of measurement noise explored here does not change the distribution's qualitative shape.

bioRxiv preprint doi: https://doi.org/10.1101/2024.01.03.574108; this version posted June 11, 2025. The copyright holder for this preprint (which was not certified by peer review) is the author/funder, who has granted bioRxiv a license to display the preprint in perpetuity. It is made available under aCC-BY 4.0 International license.

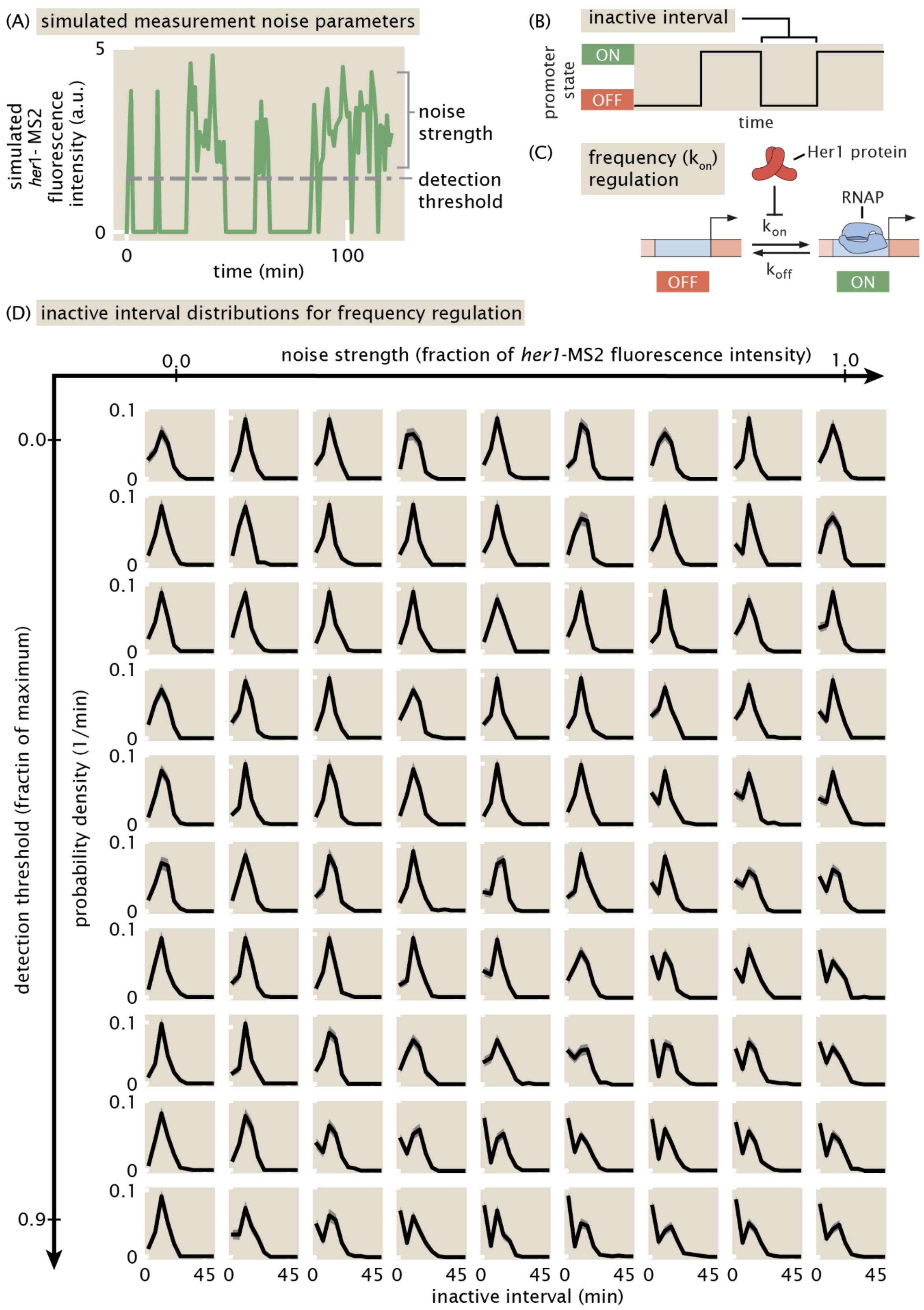

bioRxiv preprint doi: https://doi.org/10.1101/2024.01.03.574108; this version posted June 11, 2025. The copyright holder for this preprint (which was not certified by peer review) is the author/funder, who has granted bioRxiv a license to display the preprint in perpetuity. It is made available under aCC-BY 4.0 International license.

Supplemental Figure 8: Inactive interval distributions for the frequency regulation bursting model remain peaked for a wide range of simulated measurement noise parameters. Our goal is to assess the impact of the MS2 measurement process on our ability to correctly identify the shape of burst interval distributions. (A) Simulated *her1*-MS2 trace illustrating the two measurement noise parameters: noise strength, which determines the level of measurement noise within a burst, and detection threshold, which determines how strong the *her1*-MS2 signal must be to be detected. Specifically, “noise strength” is the amplitude of multiplicative Gaussian noise added to the simulated *her1*-MS2 trace; we vary the noise strength from 0.0 to 1.0. The “detection threshold” is implemented by setting all values less than the detection threshold to zero; we vary the detection threshold from 0.0 to 0.9 times the maximum *her1*-MS2 signal. Other model parameters are described in the “Generalized bursting model with feedback” section of the Methods. These parameter values were chosen to produce quasi-regular oscillations while being consistent with the literature where possible. (B) In this figure, we focus on the analysis of the inactive burst interval, defined as the duration of time the promoter is in the OFF state. (C) We also focus here on the case of frequency regulation, where Her1 proteins regulate the rate of the promoter turning off, k_{off}. With our chosen parameters and in the absence of measurement noise, the inactive interval distribution is peaked, reflecting the regulation of k_{on} that reduces stochasticity in burst initiation. (D) We explored how measurement artifacts affect the shape of this distribution by creating a 2D grid of inactive interval distributions, with increasing noise strength to the right and increasing detection threshold going down. The distribution remains peaked over a wide range of the explored parameter space, with high noise strength and/or high detection threshold adding an exponential background to the distribution.

bioRxiv preprint doi: https://doi.org/10.1101/2024.01.03.574108; this version posted June 11, 2025. The copyright holder for this preprint (which was not certified by peer review) is the author/funder, who has granted bioRxiv a license to display the preprint in perpetuity. It is made available under aCC-BY 4.0 International license.

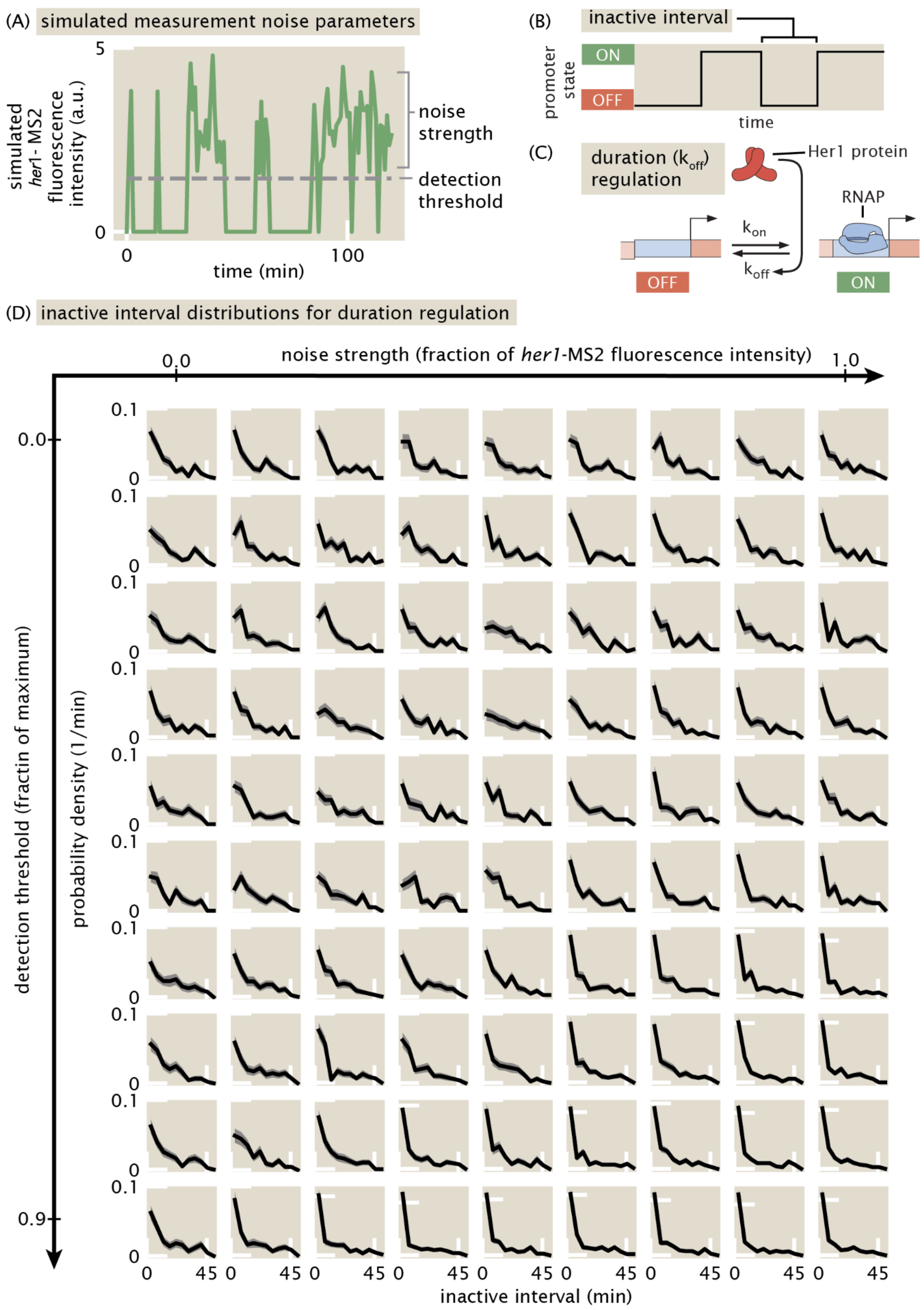

bioRxiv preprint doi: https://doi.org/10.1101/2024.01.03.574108; this version posted June 11, 2025. The copyright holder for this preprint (which was not certified by peer review) is the author/funder, who has granted bioRxiv a license to display the preprint in perpetuity. It is made available under aCC-BY 4.0 International license.

Supplemental Figure 9: Inactive interval distributions for the duration regulation bursting model remain exponential for a wide range of simulated measurement noise parameters. Our goal is to assess the impact of the MS2 measurement process on our ability to correctly identify the shape of burst interval distributions. (A) Simulated *her1*-MS2 trace illustrating the two measurement noise parameters: noise strength, which determines the level of measurement noise within a burst, and detection threshold, which determines how strong the *her1*-MS2 signal must be to be detected. Specifically, "noise strength" is the amplitude of multiplicative Gaussian noise added to the simulated *her1*-MS2 trace; we vary the noise strength from 0.0 to 1.0. The "detection threshold" is implemented by setting all values less than the detection threshold to zero; we vary the detection threshold from 0.0 to 0.9 times the maximum *her1*-MS2 signal. Other model parameters are described in the "Generalized bursting model with feedback" section of the Methods. These parameter values were chosen to produce quasi-regular oscillations while being consistent with the literature where possible. (B) In this figure, we focus on the analysis of the inactive burst interval, defined as the duration of time the promoter is in the OFF state. (C) We also focus here on the case of duration regulation, where Her1 proteins regulate the rate of the promoter turning off, k_{off}. In the absence of measurement noise, the inactive interval distribution for duration regulation is exponential, since the inactive interval is set by k_{on}, which is constant here. (D) We explored how measurement artifacts affect the shape of this distribution by creating a 2D grid of inactive interval distributions, with increasing noise strength to the right and increasing detection threshold going down. The distribution remains exponential over all of the explored parameter space.

bioRxiv preprint doi: https://doi.org/10.1101/2024.01.03.574108; this version posted June 11, 2025. The copyright holder for this preprint (which was not certified by peer review) is the author/funder, who has granted bioRxiv a license to display the preprint in perpetuity. It is made available under aCC-BY 4.0 International license.

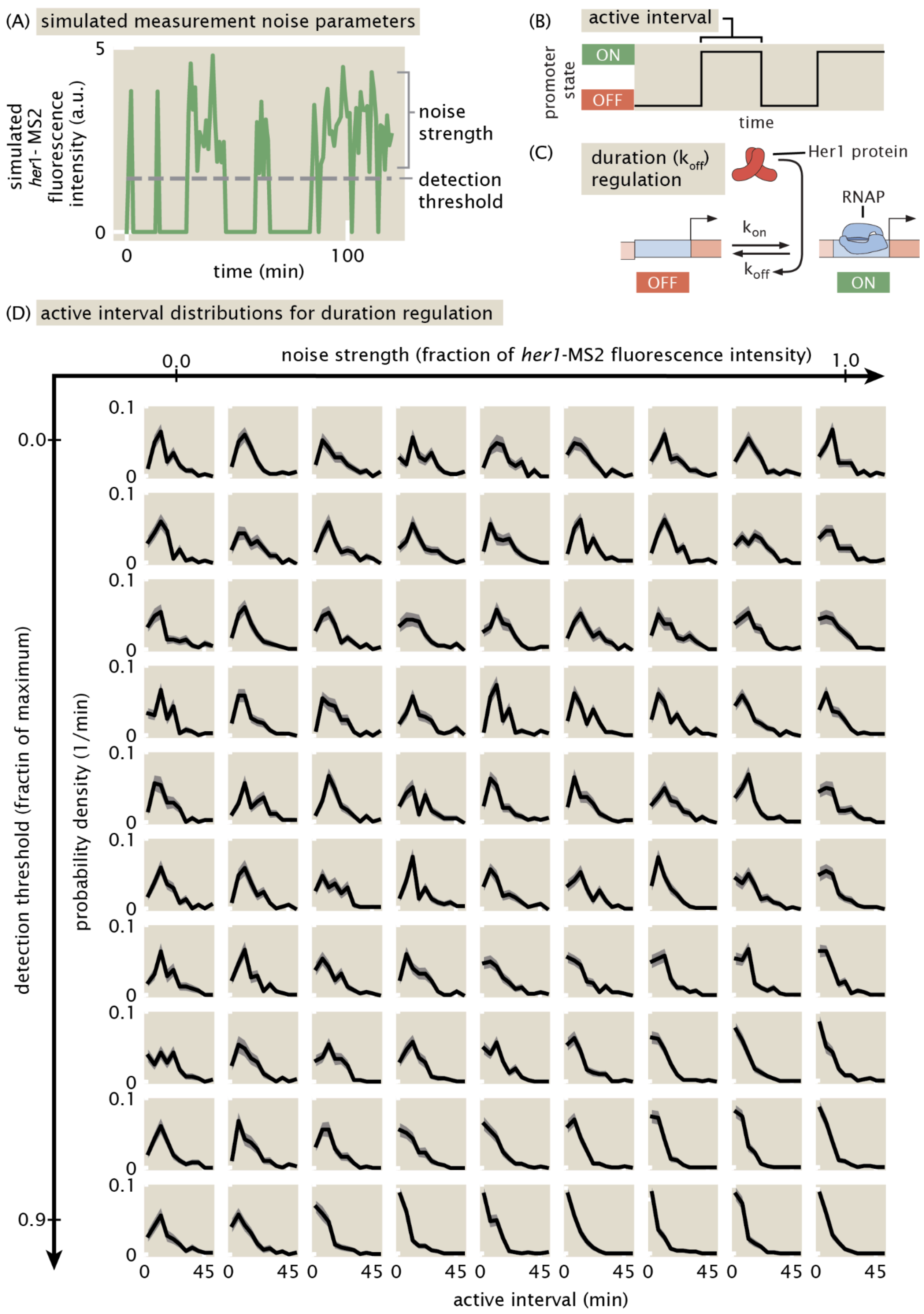

bioRxiv preprint doi: https://doi.org/10.1101/2024.01.03.574108; this version posted June 11, 2025. The copyright holder for this preprint (which was not certified by peer review) is the author/funder, who has granted bioRxiv a license to display the preprint in perpetuity. It is made available under aCC-BY 4.0 International license.

Supplemental Figure 10: Active interval distributions for the duration regulation bursting model remain peaked for a wide range of simulated measurement noise parameters. (A) Our goal is to assess the impact of the MS2 measurement process on our ability to correctly identify the shape of burst interval distributions. (A) Simulated *her1*-MS2 trace illustrating the two measurement noise parameters: noise strength, which determines the level of measurement noise within a burst, and detection threshold, which determines how strong the *her1*-MS2 signal must be to be detected. Specifically, "noise strength" is the amplitude of multiplicative Gaussian noise added to the simulated *her1*-MS2 trace; we vary the noise strength from 0.0 to 1.0. The "detection threshold" is implemented by setting all values less than the detection threshold to zero; we vary the detection threshold from 0.0 to 0.9 times the maximum *her1*-MS2 signal. Other model parameters are described in the "Generalized bursting model with feedback" section of the Methods. These parameter values were chosen to produce quasi-regular oscillations while being consistent with the literature where possible. (B) In this figure, we focus on the analysis of the active burst interval (also known as burst duration), defined as the duration of time the promoter is in the ON state. (C) We also focus here on the case of duration regulation, where Her1 proteins regulate the rate of the promoter turning off, k_{off}. With our chosen parameters and in the absence of measurement noise, the active interval distribution for duration regulation is peaked, reflecting the regulation of k_{off} that reduces stochasticity in burst duration. (D) We explored how measurement artifacts affect the shape of this distribution by creating a 2D grid of active interval distributions, with increasing noise strength to the right and increasing detection threshold going down. The distribution remains peaked over a wide range of the explored parameter space, with high noise strength and/or high detection threshold adding an exponential background to the distribution that eventually dominates.

bioRxiv preprint doi: https://doi.org/10.1101/2024.01.03.574108; this version posted June 11, 2025. The copyright holder for this preprint (which was not certified by peer review) is the author/funder, who has granted bioRxiv a license to display the preprint in perpetuity. It is made available under aCC-BY 4.0 International license.

Supplemental Movies

Supplemental Movie 1: Maximum intensity projections of AiryScan confocal microscopy images of zebrafish embryo with nuclei shown in red and *her1*-MS2 in green. Due to cell motion, manual adjustment of the microscope stage was required every few minutes to keep the same cells in the field of view. These manual adjustments correspond to the discrete jumps in the image observed throughout the movie.

Supplemental Movie 2: 3D rendering of light sheet fluorescence microscopy images of a zebrafish embryo with nuclei false colored according to the label assigned to them by the nuclear tracking algorithm. To better resolve somites, skin nuclei were computationally removed. Some flickering in the movie occurs due to imperfect segmentation of the skin. Aside from this surface-level flickering, the overall stability of colors conveys the high level of tracking accuracy.

Supplemental Movie 3: Rotating 3D rendering of light sheet fluorescence microscopy images of a zebrafish embryo with nuclei false colored according to the label assigned to them by the nuclear tracking algorithm. To better resolve somites, skin nuclei were computationally removed. Some flickering in the movie occurs due to imperfect segmentation of the skin. Aside from this surface-level flickering, the overall stability of colors conveys the high level of tracking accuracy.

Supplemental Movie 4: 3D rendering of light sheet fluorescence microscopy images of a zebrafish embryo with nuclei shown in gray and false-colored in proportion to their *her1*-MS2 signal.

Supplemental Movie 5: Rotating 3D rendering of light sheet fluorescence microscopy images of a zebrafish embryo with nuclei shown in gray and false-colored in proportion to their *her1*-MS2 signal.

Supplemental Movie 6: Animated 3D rendering of light sheet fluorescence microscopy images of a zebrafish embryo with nuclei shown in gray and false-colored in proportion to their *her1*-MS2 signal (left) and in proportion to their predicted protein signal (right). See Methods for details of protein prediction.

Supplemental Movie 7: Animated 3D rendering of light sheet fluorescence microscopy images of a zebrafish embryo with nuclei shown in magma and the anterior-posterior axis, defined through a combination of manual labeling and spline interpolation, shown in white spheres.

Appendix

bioRxiv preprint doi: https://doi.org/10.1101/2024.01.03.574108; this version posted June 11, 2025. The copyright holder for this preprint (which was not certified by peer review) is the author/funder, who has granted bioRxiv a license to display the preprint in perpetuity. It is made available under aCC-BY 4.0 International license.

See Supplemental Appendix File

Supplemental Data Files

Supplemental Data File 1: Dataset_1.pkl. *pandas* DataFrame that is the output of the image analysis pipeline (images from the Dorado light sheet microscope). Each row corresponds to an MS2 spot. Columns contain various spot features. See the code for details. This dataset was used for Figures 3 and 4.

Supplemental Data File 2: Dataset_1_Curated.pkl. *pandas* DataFrame that is the output of the image analysis pipeline (from the Dorado light sheet microscope) and manually curated, with each spot visually confirmed and linked to the correct nucleus. Each row corresponds to an MS2 spot. Columns contain various spot features. See the code for details. This dataset was used for the single-cell analysis in Figures 5 and 6.

Supplemental Data File 3: Non_Blank_Time_Points.pkl. *numpy* array that contains the true scan numbers of each time point in the Dorado dataset. As discussed in the Methods section, hardware communication issues during acquisition led to missing images for ~10% of scans. These blank time points were removed from the image dataset, but the proper timing information is stored in the non_blank_timepoints array.

Supplemental Data File 4: Dataset_1_Nuclear_Tracks.csv. *.csv* file that contains the location and identity of every tracked nucleus.

Supplemental Data File 5: Simulated_Intervals.pkl. Pickled lists containing the simulated active and inactive intervals used in Figure 6. The order of the data is: amplitude regulation, frequency regulation, duration regulation, frequency and duration regulation. For each regulation mode there are 3 lists: active intervals, inactive intervals, and periods. See also the corresponding Fig. 6 notebook on the paper’s github repository for the simulation code.
