# Supplementary material for: Single-cell transcriptional dynamics in a living vertebrate
Source: bioRxiv. 2024 Jan 4:2024.01.03.574108. Preprint. [Version 1] doi: 10.1101/2024.01.03.574108 (PMC10802376; doi:10.1101/2024.01.03.574108)
Supplement: Supplementary file 1 [file NIHPP2024.01.03.574108v1-supplement-9.pdf]

bioRxiv preprint doi: https://doi.org/10.1101/2024.01.03.574108; this version posted January 5, 2024. The copyright holder for this preprint (which was not certified by peer review) is the author/funder, who has granted bioRxiv a license to display the preprint in perpetuity. It is made available under aCC-BY 4.0 International license.

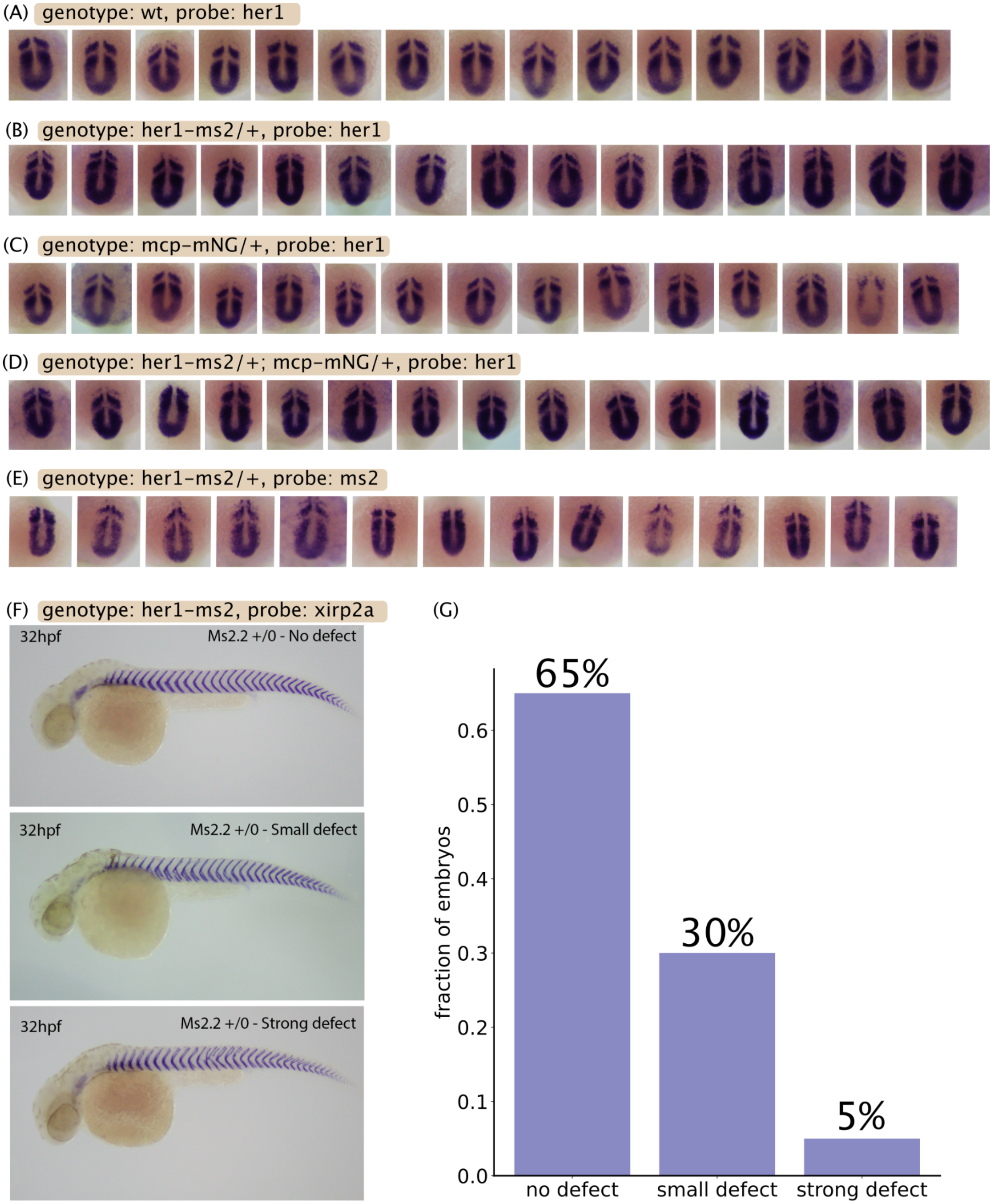

Supplemental Figure 1: Further controls show overall healthy somitogenesis of *her1*-MS2 and MCP-mNeonGreen transgenic lines. (A)-(D): In situ hybridization assays against *her1* and MS2 in various genotypes at the 12-14 somite stage embryos showing qualitative

bioRxiv preprint doi: https://doi.org/10.1101/2024.01.03.574108; this version posted January 5, 2024. The copyright holder for this preprint (which was not certified by peer review) is the author/funder, who has granted bioRxiv a license to display the preprint in perpetuity. It is made available under aCC-BY 4.0 International license.

agreement of the *her1* expression pattern. All embryos are offspring of a cross of *her1*-MS2 and MCP-mNeonGreen heterozygous animals. (A) Wild-type embryos lacking both markers for MS2 and MCP show normal *her1* expression patterns. (B) “her1-ms2” embryos carrying only the MS2 transgene show healthy *her1* expression. (C) “mcp-mNG” embryos carrying only the MCP transgene show healthy *her1* expression. (D) Embryos carrying both transgenes show healthy *her1* expression. (E) In situ hybridization assay against the MS2 sequence in *her1*-MS2 embryos showing that the reporter qualitatively recapitulates the endogenous *her1* expression pattern. (F) *In situ* hybridization assay for the somite boundary marker *xirp2a*, showing examples of no defect, small defect, strong defect. When defects do occur, they are limited to the trunk region, which completes somite formation before our imaging experiments begin and is thus not included in our analysis. (G) The majority of animals show no defects at 32 hours post fertilization.

bioRxiv preprint doi: https://doi.org/10.1101/2024.01.03.574108; this version posted January 5, 2024. The copyright holder for this preprint (which was not certified by peer review) is the author/funder, who has granted bioRxiv a license to display the preprint in perpetuity. It is made available under aCC-BY 4.0 International license.

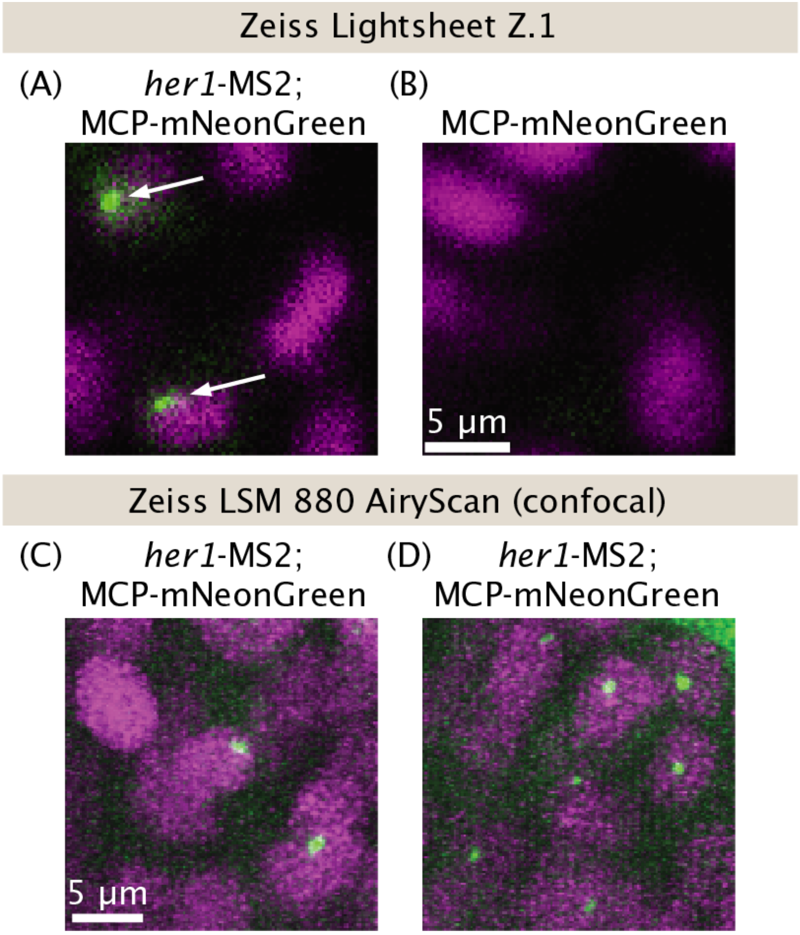

Supplemental Figure 2: Characterization of spots on commercial light sheet and confocal fluorescence microscopes. (A,B) Spots (white arrows) are detected on the Zeiss Z.1 Lightsheet but only when the reporter gene is present. MCP-mNeonGreen fish show no puncta in the absence of the *her1*-MS2 reporter. Single z-slices of MCP-mNeonGreen (green); h2B-mScarlet (magenta) embryos (A) with *her1*-MS2 or (B) without *her1*-MS2. (C)-(D) Two examples of spots on a Zeiss LSM 880 confocal microscope with AiryScan processing. Contrast was adjusted manually such that background MCP levels were approximately equal in all images.

bioRxiv preprint doi: https://doi.org/10.1101/2024.01.03.574108; this version posted January 5, 2024. The copyright holder for this preprint (which was not certified by peer review) is the author/funder, who has granted bioRxiv a license to display the preprint in perpetuity. It is made available under aCC-BY 4.0 International license.

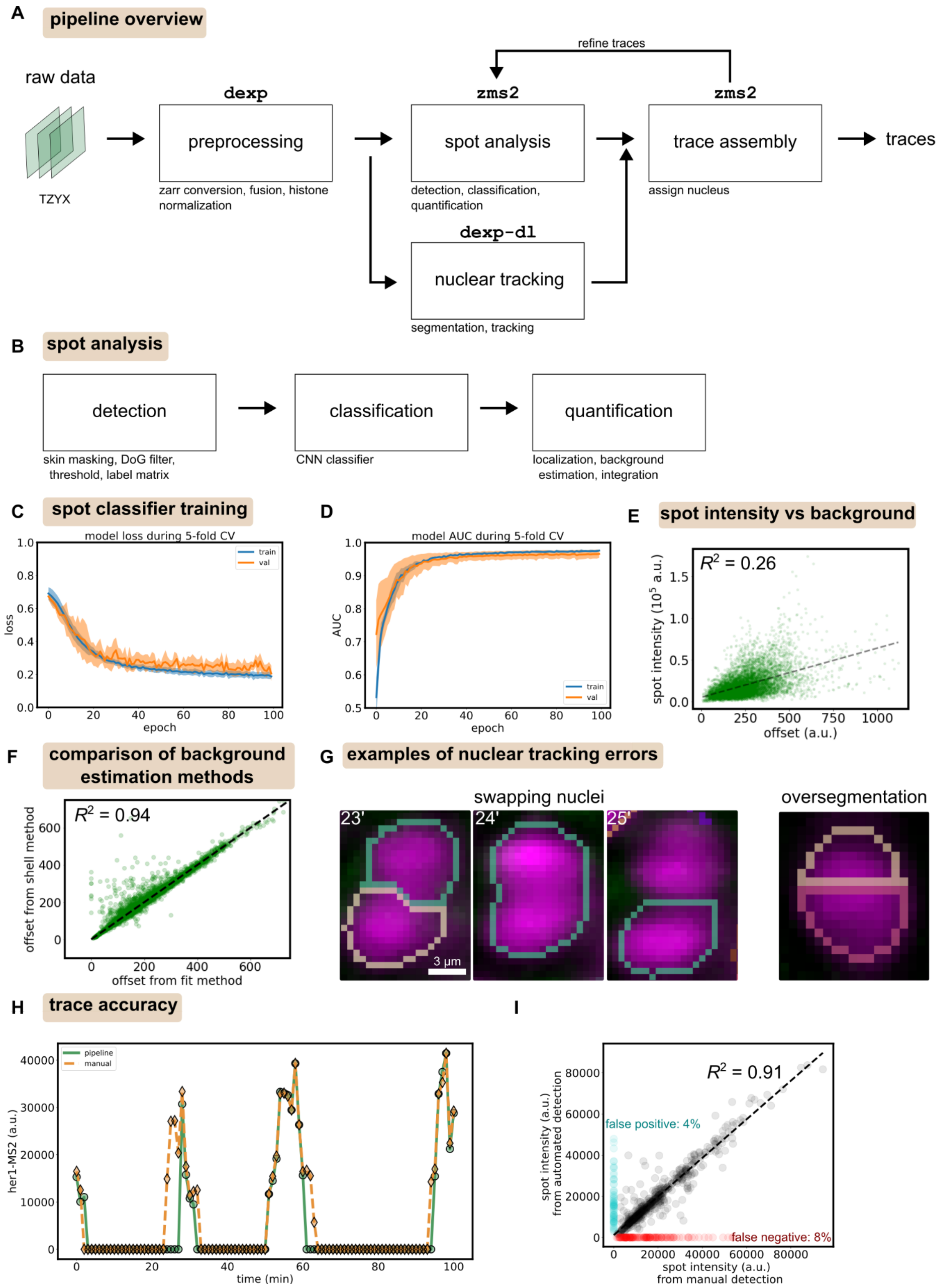

bioRxiv preprint doi: https://doi.org/10.1101/2024.01.03.574108; this version posted January 5, 2024. The copyright holder for this preprint (which was not certified by peer review) is the author/funder, who has granted bioRxiv a license to display the preprint in perpetuity. It is made available under aCC-BY 4.0 International license.

Supplemental Figure 3: Overview of image analysis pipeline and its accuracy. (A) Schematic of the full pipeline. The pipeline takes 4D image stacks as input, one for the nuclear marker and one for the MS2-MCP reporter per light sheet view. Preprocessing using the *dexp* package involves conversion to the zarr file format, fusion of multiple light sheet views (if necessary), and normalization of the nuclear marker channel to prepare for nuclear segmentation. After preprocessing, the nuclear and spot channels are split, with the nuclear channel undergoing segmentation and tracking via our *dexp-dl* code and the spot channel passing through our spot pipeline, called *zms2*, which detects and analyzes MS2 spots. The outputs of these packages are combined into traces. Traces are then refined using an iterative computational algorithm. In brief, the algorithm identifies abrupt gaps in traces and attempts to fill them in by extracting a voxel around the expected spot location, passing it back through the classification and quantification modules, and seeing if the spot passes a set of criteria (Methods). (B) Schematic spot analysis section of the full pipeline. Candidate spots are detected and then classified as a true spot or a false-positive detection using a binary CNN classifier. The culled list of spots then undergo quantification, where the total fluorescence intensity of the signal is computed. DoG=Difference of Gaussians, CNN=Convolutional Neural Network. Skin masking=computational removal of bright and irrelevant skin cells. Thresholding occurs on the DoG-filtered image to create a binary mask, which is transformed to a label matrix that assigns a unique identifier to each candidate spot. (C) Binary cross-entropy loss function of convolutional neural network spot classifier over training shows that the model converged with minimal overfitting. Solid lines are means and shaded error bars standard deviations over 5 folds of cross validation. Blue=training set, Orange=validation set. (D) Area Under the Curve (AUC) during training. (E) Spot intensity vs. background level (“offset”) showing an extremely weak correlation (R^2=0.26), which is indicative of MCP saturation and reliable measures of absolute fluorescence intensity. (F) Comparison of two methods for background estimation, described in the Methods section. “Shell” refers to taking the mean of pixel values in a shell around the spot. “Fit” refers to fitting a Gaussian with an offset. We find that the two methods give very similar results, with the data clustering closely around the 1:1 line (black dashed line) and an R^2 of 0.94. The best fit slope is 1.049 +/- 0.003. (G) Examples of nuclear tracking errors, including when the algorithm swaps the identities of nearby nuclei (left), and when it splits a single nucleus into two segments (right). Shown are single z-slices of histones (magenta), with the algorithm-assigned identity of each nucleus marked by a unique colored outline. MCP-mNeonGreen background is faintly visible in green. (H) Example comparison of a pipeline-derived trace (green solid line) and the corresponding manually-derived trace (orange dashed line), obtained by manually clicking on spots in images and passing these spots through the quantification pipeline. (I) Comparison of spot intensities between pipeline and manual traces across 728 spots from 26 nuclei. Cases where both methods detect a spot are colored in black. Linear regression to these points shows strong correlation (R^2 = 0.91). Cases in which a human detected a spot and the pipeline did not (false negative) are colored in red. These cases are 8% of all spots. Cases in which a human did not detect a spot but the pipeline did (false positive) are colored in cyan. These cases are 4% of all spots.

bioRxiv preprint doi: https://doi.org/10.1101/2024.01.03.574108; this version posted January 5, 2024. The copyright holder for this preprint (which was not certified by peer review) is the author/funder, who has granted bioRxiv a license to display the preprint in perpetuity. It is made available under aCC-BY 4.0 International license.

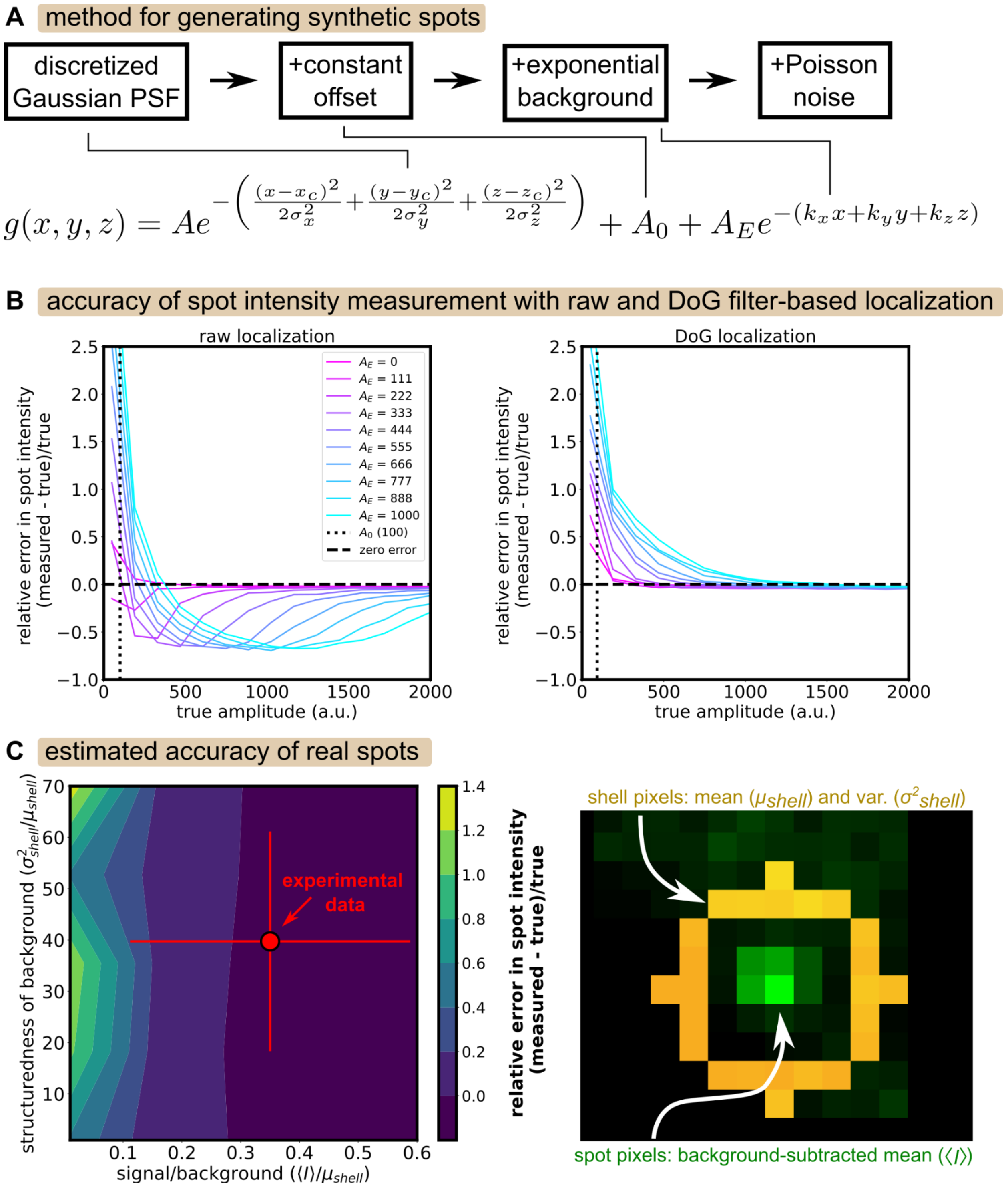

Supplemental Figure 4: Characterization of spot intensity measurement accuracy using synthetic spots. (A) Schematic of method for generating synthetic spots with structured background. The PSF is modeled as a Gaussian function, to which we add a constant offset and an exponential background, the latter being a simple model of the structured MCP background

bioRxiv preprint doi: https://doi.org/10.1101/2024.01.03.574108; this version posted January 5, 2024. The copyright holder for this preprint (which was not certified by peer review) is the author/funder, who has granted bioRxiv a license to display the preprint in perpetuity. It is made available under aCC-BY 4.0 International license.

we observe in real spots. The wavevector, *k*, of the background is taken to have a length of 10 pixels (roughly the length of the spot in voxels) and a random direction. The pixels of the final image are then drawn from a Poisson distribution with mean equal to $g(x,y,z)$. (B) Relative error in spot intensity (measured - true)/true vs true spot amplitude (the parameter *A* in the equation in panel (A)) for different strengths of the exponential background (via the parameter A_E). Left: spot localization via Gaussian fitting of the raw image. Right: spot localization via Gaussian fitting of a Difference-of-Gaussians (DoG)-filtered image. After localization, background estimation is done with the "shell" method described in the Methods. Intensity is then the sum of background-subtracted pixels inside a defined ellipsoid. Intensity errors are larger when localization is done on the raw pixels, due to the structured background. (C) Left: Placing the experimental data (red circle=mean, red bars=standard deviation across all spots) in a regime diagram describing relative accuracy of the intensity measurement (heatmap) as a function of signal/background and "structuredness" of the background. The x-axis, signal/background, is the average background-subtracted value of spot pixel intensities, i.e., 0.1 = 10% above background. The y-axis, structuredness of background, is the variance of shell pixels divided by the mean of shell pixels. For a uniform background with Poisson noise, the structuredness will be 1. The experimental data has a background with structuredness of approximately 40 on average. The simulations from (B) were binned into a 2D histogram according to their signal/background and structuredness levels to create the final heatmap of relative error. This heatmap illustrates that, based on our model of spots, the DoG-based localization method should lead to highly accurate intensity estimations, with a relative error of less than 10%. Right: Schematic of the parameters used to define the axes of the heatmap on the left, using a single z slice of a real spot (green) with shell pixels highlighted in orange.

bioRxiv preprint doi: https://doi.org/10.1101/2024.01.03.574108; this version posted January 5, 2024. The copyright holder for this preprint (which was not certified by peer review) is the author/funder, who has granted bioRxiv a license to display the preprint in perpetuity. It is made available under aCC-BY 4.0 International license.

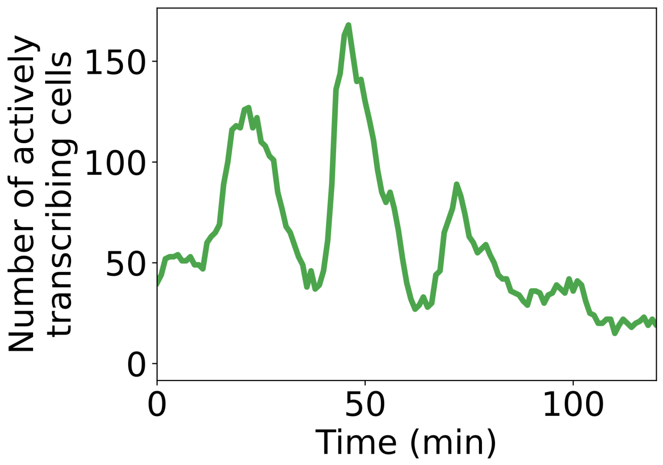

Supplemental Figure 5: Tissue-wide transcriptional activity. The total number of cells with detected *her1*-MS2 spots oscillates over time with a period of approximately 25 min, similar to the somitogenesis period.

bioRxiv preprint doi: https://doi.org/10.1101/2024.01.03.574108; this version posted January 5, 2024. The copyright holder for this preprint (which was not certified by peer review) is the author/funder, who has granted bioRxiv a license to display the preprint in perpetuity. It is made available under aCC-BY 4.0 International license.

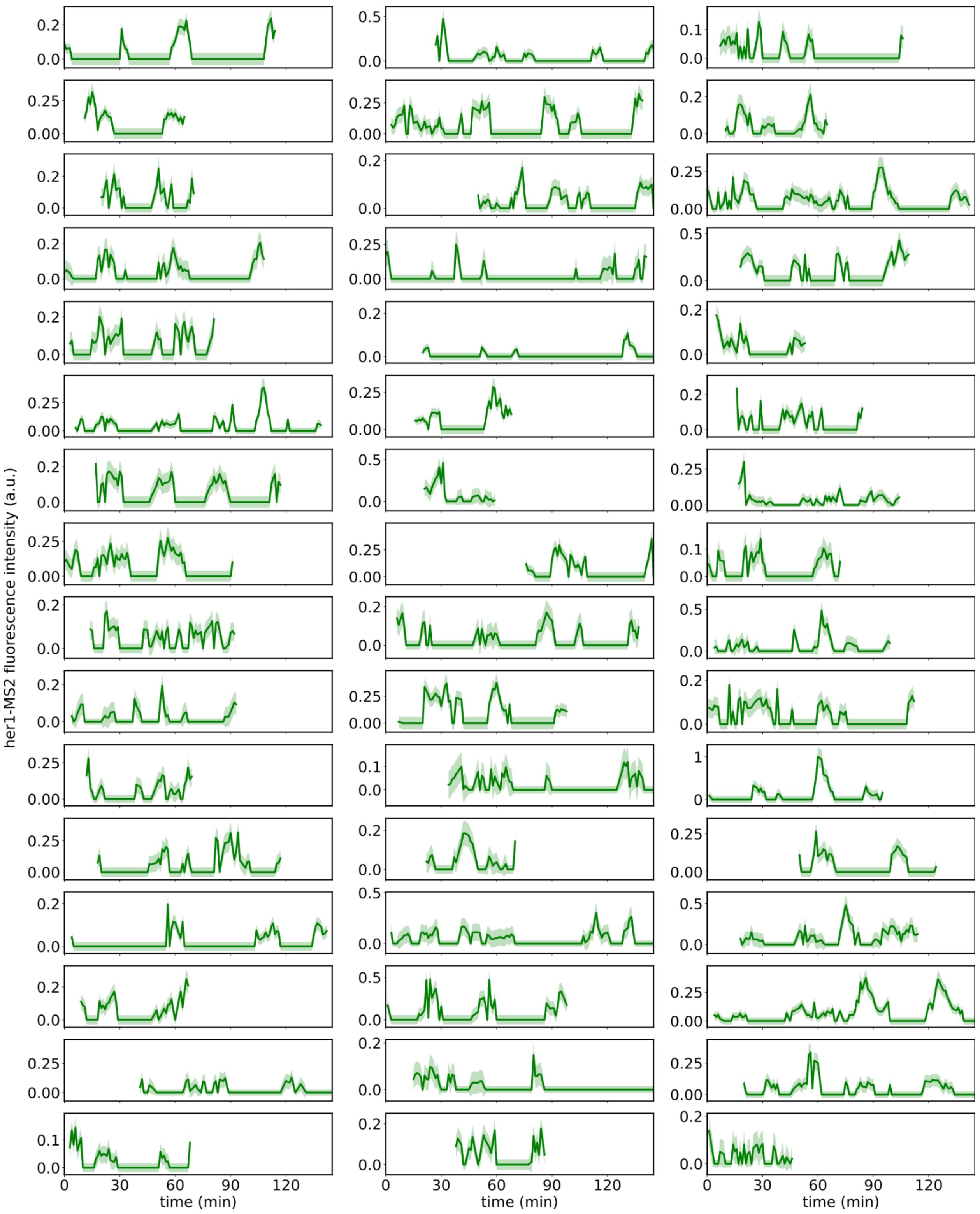

Supplemental Figure 6. 48 randomly chosen traces with at least 20 spots per trace.

bioRxiv preprint doi: https://doi.org/10.1101/2024.01.03.574108; this version posted January 5, 2024. The copyright holder for this preprint (which was not certified by peer review) is the author/funder, who has granted bioRxiv a license to display the preprint in perpetuity. It is made available under aCC-BY 4.0 International license.

Supplemental Movie Captions

Supplemental Movie 1: Maximum intensity projections of AiryScan confocal microscopy images of zebrafish embryo with nuclei shown in red and *her1*-MS2 in green. Due to cell motion, manual adjustment of the microscope stage was required every few minutes to keep the same cells in the field of view. These manual adjustments correspond to the discrete jumps in the image observed throughout the movie.

Supplemental Movie 2: 3D rendering of light sheet fluorescence microscopy images of a zebrafish embryo with nuclei false colored according to the label assigned to them by the nuclear tracking algorithm. To better resolve somites, skin nuclei were computationally removed. Some flickering in the movie occurs due to imperfect segmentation of the skin. Aside from this surface-level flickering, the overall stability of colors conveys the high level of tracking accuracy.

Supplemental Movie 3: 3D rendering of light sheet fluorescence microscopy images of a zebrafish embryo with nuclei shown in gray and false-colored in proportion to their *her1*-MS2 signal.

Supplemental Movie 4: Rotating 3D rendering of light sheet fluorescence microscopy images of a zebrafish embryo with nuclei shown in gray and false-colored in proportion to their *her1*-MS2 signal.

Supplemental Movie 5: Animated 3D rendering of light sheet fluorescence microscopy images of a zebrafish embryo with nuclei shown in gray and false-colored in proportion to their *her1*-MS2 signal (left) and in proportion to their predicted protein signal (right). See Methods for details of protein prediction.

Supplemental Movie 6: Animated 3D rendering of light sheet fluorescence microscopy images of a zebrafish embryo with nuclei shown in magma and the anterior-posterior axis, defined through a combination of manual labeling and spline interpolation, shown in white spheres.

Supplemental Data Files

Supplemental Data File 1: Dataset_1.pkl. *pandas* DataFrame that is the output of the image analysis pipeline for Dataset 1 (from the Dorado light sheet microscope). Each row corresponds to an MS2 spot. Columns contain various spot features. See the code for details. This dataset was used for the majority of the analysis in this paper.

Supplemental Data File 2: Dataset_2.pkl. *pandas* DataFrame that is the output of the image analysis pipeline for Dataset 2 (from the Zeiss light sheet microscope). Each row corresponds to an MS2 spot. Columns contain various spot features. See the code for details. This dataset was used only for the analysis of the number of transcriptional bursts per predicted protein oscillation.

bioRxiv preprint doi: https://doi.org/10.1101/2024.01.03.574108; this version posted January 5, 2024. The copyright holder for this preprint (which was not certified by peer review) is the author/funder, who has granted bioRxiv a license to display the preprint in perpetuity. It is made available under aCC-BY 4.0 International license.

Supplemental Data File 3: Dataset_1_Manual.pkl. *pandas* DataFrame that is the output of the image analysis pipeline for the manually-curated Dataset 1 (from the Dorado light sheet microscope). Spots were identified in images manually by dropping points in *napari*. Voxels around these points were then fed through the quantification module (with no filling in). Each row corresponds to an MS2 spot. Columns contain various spot features. See the code for details. This dataset was used to complement the analysis of number of transcriptional bursts per predicted protein oscillation and for plotting traces in Fig.5.

Supplemental Data File 4: Dataset_2_Manual.pkl. *pandas* DataFrame that is the output of the image analysis pipeline for the manually-curated Dataset 2 (from the Zeiss light sheet microscope). Spots were identified in images manually by dropping points in *napari*. Voxels around these points were then fed through the quantification module (with no filling in). Each row corresponds to an MS2 spot. Columns contain various spot features. See the code for details. This dataset was used to complement the analysis of number of transcriptional bursts per predicted protein oscillation.

Supplemental Data File 5: Non_Blank_Time_Points.pkl. *numpy* array that contains the true scan numbers of each time point in the Dorado dataset. As discussed in the Methods section, hardware communication issues during acquisition led to missing images for ~10% of scans. These blank time points were removed from the image dataset, but the proper timing information is stored in the non_blank_timepoints array.

Supplemental Data File 6: Dataset_1_Nuclear_Tracks.csv. *.csv* file that contains the location and identity of every tracked nucleus.
