# Supplementary material for: Single-cell transcriptional dynamics in a living vertebrate
Source: bioRxiv. 2024 Jan 4:2024.01.03.574108. Preprint. [Version 1] doi: 10.1101/2024.01.03.574108 (PMC10802376; doi:10.1101/2024.01.03.574108)
Supplement: Supplementary file 2 [file media-1.pdf]

Appendix for "Single-cell transcriptional dynamics in a living vertebrate"

Elizabeth Eck[*1] Bruno Moretti[*2,5] Brandon H. Schlomann[*2]
Jordão Bragantini[5] Merlin Lange[5] Xiang Zhao[5]
Shruthi VijayKumar[5] Cristina Loureiro[6] Guillaume Valentin[6],
Daniele Soroldoni[6&] Loïc A. Royer[5] Andrew C. Oates[6,7,8]
Hernan G. Garcia[2,3,4,5+]

January 2, 2024

1. Biophysics Graduate Group, University of California at Berkeley, Berkeley, USA
2. Department of Molecular and Cell Biology, University of California, Berkeley, CA, USA
3. Department of Physics, University of California, Berkeley, CA, USA
4. Institute for Quantitative Biosciences-QB3, University of California, Berkeley, CA, USA
5. Chan Zuckerberg Biohub – San Francisco, San Francisco, CA, USA
6. Institute of Bioengineering, EPFL; Lausanne, CH
7. Department of Cell and Developmental Biology, UCL; London, UK
8. The Francis Crick Institute; London, UK

[*]Equal contributors. Author order is alphabetical.
[+]Corresponding author: hggarcia@berkeley.edu
[&]Present address: Vienna BioCenter, Vienna, Austria

1 Predicting protein signal from MS2 signal

An MS2 signal is an approximation of the instantaneous mRNA production rate. Given an MS2 signal, we predict the downstream protein signal by assuming the following flow of information, from MS2 (x) to total mRNA (m) to total protein (p):

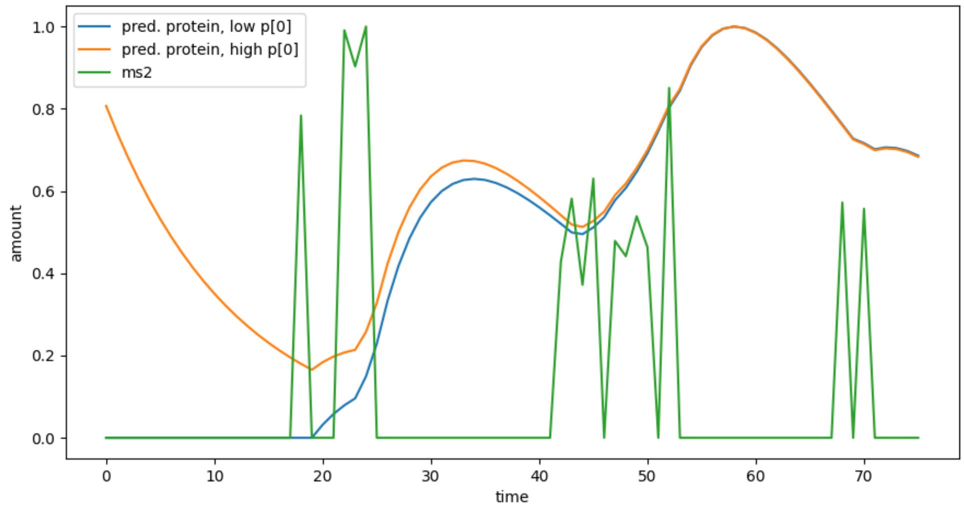

Figure 1: Predicted protein traces for different initial conditions converge to the same solution. Green = raw MS2 trace. Blue = predicted protein curve starting from low initial protein. Orange = predicted protein curve starting from high initial protein. Parameters: $\gamma_m = 0.1$ min^{-1}, $\gamma_p = 0.08$ min^{-1}. Each trace was rescaled to have a maximum of 1.

$$x = \text{given} \tag{1}$$
$$\dot{m} = r_m x - \gamma_m m \tag{2}$$
$$\dot{p} = r_p m - \gamma_p p. \tag{3}$$

Here, the rs are production rates, and γs are decay rates. Given an MS2 signal and this system of equations, the predicted protein is obtained by forward integration.

The problem is that the initial conditions for mRNA and protein are unknown. Here, we show that the effect of the initial condition decays exponentially with a rate equal to the total decay rate. Regardless of initial conditions, in this linear system the predicted protein and mRNA levels will converge to the same value on the timescale of decay. An example of this phenomenon is shown in Fig. 1 for a real MS2 trace. Therefore, the prudent thing to do is to pick an arbitrary initial condition, but then throw out the predictions for mRNA and protein up to this timescale—we simply don't have the information to predict what those levels are.

1.1 Solving the system of equations using the Laplace transform

We show the effect of initial condition using the Laplace transform:

$$\mathcal{L}(x(t))(s) \equiv \hat{x}(s) = \int_0^\infty dt\, \mathrm{e}^{-st} x(t). \tag{4}$$

We restrict our derivation to predicting only the total mRNA signal; extension to protein is straightforward.

Recall the relation between Laplace transform and differentiation:

$$\mathcal{L}\left(\frac{dx}{dt}(t)\right)(s) = s\hat{x} - x(t=0). \tag{5}$$

Using this property, we can Laplace transform the equation for m above:

$$s\hat{m} - m(t=0) = r_m \hat{x} - \gamma_m \hat{m}. \tag{6}$$

Let's define $m(t=0) \equiv m_0$ and solve for $\hat{m}(s)$:

$$\hat{m}(s) = \frac{m_0}{s+\gamma_m} + r_m \frac{\hat{x}}{s+\gamma_m}. \tag{7}$$

The solution has 2 pieces, one coming from the initial condition, and one coming from the driving MS2 signal x. We now need to invert the Laplace transform to solve for $m(t)$. A well-known Laplace transform-inverse transform pair is

$$\mathrm{e}^{-\gamma t} \Longleftrightarrow \frac{1}{s+\gamma}, \tag{8}$$

where the double arrow indicates the Laplace transform to the right and inverse Laplace transform to the left. With this result, the first term for $\hat{m}(s)$ gives an exponential:

$$\mathcal{L}^{-1}\left(\frac{m_0}{s+\gamma_m}\right) = m_0 \mathrm{e}^{-\gamma_m t}. \tag{9}$$

The second term can be viewed as a product of two terms, $(s+\gamma_m)^{-1}$ and $\hat{x}$. Like in Fourier space, in Laplace space the product of two Laplace transforms corresponds to convolution in real space. So the inverse transform of the right hand side is the convolution of the inverse transforms of each of the terms:

$$\mathcal{L}^{-1}\left(r_m \frac{\hat{x}}{s+\gamma_m}\right) = r_m \mathrm{e}^{-\gamma_m t} \star x(t). \tag{10}$$

Putting these together we have the solution

$$m(t) = m_0 \mathrm{e}^{-\gamma_m t} + r_m \mathrm{e}^{-\gamma_m t} \star x(t). \tag{11}$$

To be specific, the convolution term is actually an incomplete convolution, in that it only goes up to time t:

$$\boxed{m(t) = m_0 \mathrm{e}^{-\gamma_m t} + r_m \int_0^t dt' x(t') \mathrm{e}^{-\gamma_m (t-t')}.} \tag{12}$$

This upper limit reflects the causality of the differential equation, i.e., mRNA at time t is only determined by MS2 signal up to time t.

So to interpret the solution: there are two parts. The core part is a convolution with a damped exponential of rate γ_m. This part reflects the low-pass filtering done by the process of accumulating and destroying mRNA. The second part reflects the initial condition, and we see explicitly that this part decays to zero exponentially with the same rate γ_m. This result proves that as $t \gg 1/\gamma_m$, the solution converges to a function that is independent of the initial condition.

Extending this result to the protein is straightforward—we now just have the two decay timescales at work, so the effect of the initial conditions fade after $1/\gamma_m + 1/\gamma_p$.

2 MS2 trace uncertainty

2.1 Uncertainty due to background fluctuations

We quantify spot intensity by finding the spot center, estimating the background intensity levels around the spot, subtracting this background from each spot pixel (enforcing non-negativity), and then summing the pixels contained in an area defined by the spot size (i.e., by the point spread function).

Mathematically, the total spot intensity, I, is given by a sum of individual pixel intensities, I_j minus the background, B:

$$I = \sum_{j \in \mathcal{V}} (I_j - B), \tag{13}$$

where the sum is over pixels contained in a neighborhood volume $\mathcal{V}$ around the spot center. Let there be N pixels in $\mathcal{V}$. Then we can write I as

$$I = \left(\sum_{j=1}^{N} I_j \right) - NB. \tag{14}$$

If we assume that the fluctuations in each pixel level, I_j, are all independent from one another (as is the typical model of shot noise in a microscope), and are independent from the background level, B, then the uncertainty in our measurement of the total spot intensity, I, is given by,

$$\sigma_I^2 = \sum_{j=1}^{N} \sigma_{I_j}^2 + N^2 \sigma_B^2. \tag{15}$$

The assumption that is typically made [1] is that the σ_{I_j}s are all the same and are equal to σ_B: that there is one typical scale of fluorescence fluctuations that affect the background and signal identically. Given this assumption, one then estimates σ_B by looking at the fluctuations in the background. This is done by fitting a smooth trend to the time trace of the background signal (where the background level is obtained, for instance, by Gaussian fitting), and then measuring the RMS deviation of the background from this trend (Fig. 2).

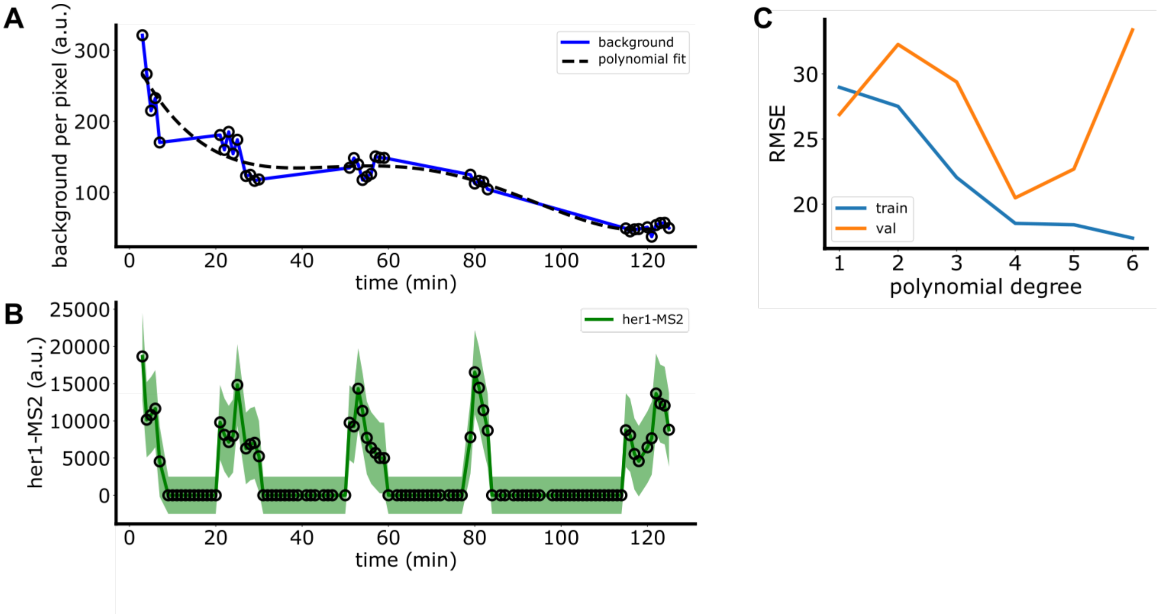

Figure 2: **Example of background fluctuation measurement.** (A) The mean background per pixel plotted over time for a given trace. Dashed line shows a polynomial fit of degree 4. (B) her1-MS2 trace with error bars derived from the background fluctuations as well as spot detection. (C) Root-mean-squared-error (RMSE) of the polynomial fit from (A) in a 5-fold cross-validation scheme, showing the train (blue) and validation (orange) errors. The minimum validation error occurs at polynomial degree 4.

With this estimate of σ_B, we get

$$\sigma_I^2 = \sum_{j=1}^{N} \sigma_B^2 + N^2\sigma_B^2 = \sigma_B^2(N + N^2). \tag{16}$$

Rearranging, we arrive at

$$\sigma_I = \sigma_B N \left(1 + \frac{1}{N}\right)^{1/2}. \tag{17}$$

Note that this differs slightly from the estimate in the work of Garcia et al.[1]:

$$\sigma_I = \sqrt{2} N \sigma_B, \tag{18}$$

2.2 Including detection uncertainty

If we are relying on automated spot detection, there is also some additional uncertainty due to the probabilities of false positive and false negative detections. Here we incorporate those sources of uncertainty. The approach is to define a compound stochastic process that combines the probability of false positive/false negative detections with the measurement uncertainty obtained above.

Let the false positive probability by p and the false negative probability be n. These are false positive and false negative rates for a whole dataset, measured by comparing a subset of traces to manually obtained traces, i.e. ground truth for the presence/absence of a spot. We break up the analysis into the case where we are adding uncertainty to detected spots and the case where we are adding uncertainty to a "zero" (no spot).

Case 1: a spot is detected

Suppose we detect a spot and measure its intensity I and uncertainty σ_I. There is a probability p that the spot is a false detection, and that the true intensity of the trace at this time point is zero. We want to incorporate this uncertainty.

Define a binary variable

$$\eta = \begin{cases} 1 & \text{if the spot is real (probability } 1-p) \\ 0 & \text{if the spot is a false positive (probability } p). \end{cases} \tag{19}$$

Now define a stochastic variable X that combines the intensity I, which we treat as a random variable, and the hidden "truth" variable η:

$$X = \begin{cases} I & \text{if } \eta = 1 \\ 0 & \text{if } \eta = 0. \end{cases} \tag{20}$$

The full uncertainty in our measurement of the intensity at this time point is the standard deviation of X. To calculate this uncertainty, we use the law of total variance, which states that

$$\text{Var}X = \mathbb{E}[\text{Var}(X|\eta)] + \text{Var}(\mathbb{E}[X|\eta]). \tag{21}$$

The first term, the average variance, is computed as follows. If $\eta = 1$, $X = I$, and $\text{Var}X = \sigma_I^2$. If $\eta = 0$, $X = 0$, and $\text{Var}X = 0$ (the intensity of a 'no spot' is exactly 0). The average variance is then given by

$$\begin{aligned} \mathbb{E}[\text{Var}(X|\eta)] &= \text{Var}(X|\eta = 1)P(\eta = 1) + \text{Var}(X|\eta = 0)P(\eta = 0) \\ &= \sigma_I^2(1-p) + 0 * p = \sigma_I^2(1-p), \end{aligned} \tag{22}$$

where in the first line $P(\eta = 1)$ is the probability that $\eta = 1$, which is $1-p$, and analogously for the $\eta = 0$.

The second term, the variance of the averages, is similarly reasoned: if $\eta = 1$, $X = I$, and given that we only have one measurement of I, we assume $\mathbb{E}[X] = I$. If $\eta = 0$, $X = 0$, and $\mathbb{E}[X] = 0$.

For convenience, define $M := \mathbb{E}[X|\eta]$. The average of M is given by

$$\bar{M} = I(1-p) + 0 * p = I(1-p). \tag{23}$$

Now we can compute

$$\begin{aligned} \text{Var}(\mathbb{E}[X|\eta]) &= \mathbb{E}[(M - \bar{M})^2] \\ &= (I - I(1-p))^2(1-p) + (0 - I(1-p))^2 p \\ &= I^2p^2(1-p) + I^2(1-p)^2 p \\ &= I^2 p(1-p)(p + 1 - p) \\ &= I^2 p(1-p). \end{aligned} \tag{24}$$

Putting these two results together, we arrive at

$$\mathrm{Var}X = \sigma_I^2(1-p) + I^2 p(1-p), \tag{25}$$

or

$$\sigma_X = \sqrt{\sigma_I^2(1-p) + I^2 p(1-p)}. \tag{26}$$

Case 2: no spot is detected

Here, we measure a zero, but there is a probability n that we missed a true spot with non-zero intensity. We will estimate the "missing" intensity by the empirical mean of the trace, $\bar{I}$—an exact number, not a stochastic one.

The approach here mirrors case 1, but is simpler, because the "missing" value is exact. Define

$$\eta = \begin{cases} 1 & \text{if there is really no spot (probability } 1-n) \\ 0 & \text{if we missed a spot (i.e. false negative with probability } n). \end{cases} \tag{27}$$

In this case, $\mathrm{Var}(X|\eta) = 0$ for both values of η, so we simply have

$$\mathrm{Var}X = \mathrm{Var}(\mathbb{E}[X|\eta]). \tag{28}$$

Let $M := \mathbb{E}[X|\eta]$. Its average

$$\bar{M} = \bar{I}n. \tag{29}$$

Then we have

$$\begin{aligned} \mathrm{Var} &= (\bar{I} - \bar{I}n)^2 n + (\bar{I}n)^2(1-n) \\ &= \bar{I}^2(1-n)^2 n + \bar{I}^2 n^2 (1-n) \\ &= \bar{I}^2 n(1-n)(1-n+n) \\ &= \bar{I}^2 n(1-n). \end{aligned} \tag{30}$$

In terms of the final uncertainty,

$$\sigma_X = \bar{I}\sqrt{n(1-n)}. \tag{31}$$

Conclusion

Putting all these results together, we arrive at the final uncertainty of our measurement

$$\sigma = \begin{cases} \sqrt{\sigma_I^2(1-p) + I^2 p(1-p)} & I > 0 \\ \\ \bar{I}\sqrt{n(1-n)} & I = 0 \end{cases} \tag{32}$$

where σ_I is the uncertainty from background fluctuations, p is the false-positive rate of detection, n is the false-negative rate of detection, I is the intensity of the individual spot, and $\bar{I}$ is the mean intensity of the trace.

3 Generalized transcriptional bursting model with feedback

In this section we provide details on the mathematical model from Fig. 6 of the main text. The goal of the model is to provide a minimal phenomenological description of transcriptional bursting with auto-repression that is flexible enough to include a range of possible regulatory mechanisms.

3.1 Background on deterministic models of the segmentation clock

The standard approach to modeling segmentation clock oscillations [2], which predicts smooth mRNA oscillations, is to use a set of delay differential equations describing mRNA and protein concentrations, m and p, that is of the form

$$\begin{aligned} \dot{m} &= r_m f(p_\tau) - \gamma_m m & (33) \\ \dot{p} &= r_p m - \gamma_p p. & (34) \\ & & (35) \end{aligned}$$

We use the notation $p_\tau \equiv p(t-\tau)$. Here, r_m and r_p are the transcription and translation rates of mRNA and protein, respectively, and γ_m and γ_p are the decay rates of mRNA

and protein, respectively. The function $f(p)$ describes auto-regulation, with the rate of transcription modulated by protein concentration. This function typically takes the form of a decreasing Hill function,

$$f(x) = \frac{1}{K_D^n + x^n}. \tag{36}$$

The time delay in the repression term is required mathematically for the system to contain a Hopf bifurcation [2, 3]. Past the Hopf bifurcation, this dynamical system exhibits oscillatory solutions, with a period set by the sum of the delay and the molecular half-lives. As an alternative to the time delay, one can get oscillatory solutions by adding a third variable to the system, representing some intermediate state of mRNA or protein (e.g., cytoplasmic vs. nuclear mRNA) [4]. Here, we focus on the delay approach.

This delay differential equation model does not exhibit transcriptional bursting per se. Near the Hopf bifurcation, oscillations become less regular and resemble stochastic dynamics [5]. Analysis of explanted single-cells suggests that the zebrafish segmentation clock is consistent with deterministic dynamics close to this regime [5]. However, in that case, both transcription rate and protein concentration should exhibit irregularities [5], whereas we observe quasi-stochastic transcriptional bursting underlying regular protein oscillations. Thus, we sought a different model to explain this behavior.

3.2 Background on the two-state model

Our approach is to extend the canonical two-state bursting model to include feedback[6]. The two-state bursting model assumes the existence of OFF and ON promoter states, described by a binary variable $\chi \in \{0, 1\}$, with mRNA production occurring only in the ON state ($\chi = 1$). Switching between ON and OFF states is stochastic, described by probability rates k_{on} and k_{off}, respectively. In the conventional two-state model, these rates are constants. Often, one chooses to ignore count noise coming from the discrete nature of mRNA and protein molecules by describing mRNA and protein concentrations with continuous variables, m and p[6]. These continuous variables are then governed by ODEs. Thus, the only stochasticity in the system is in the random switching of the promoter state. The complete system takes the form a Piecewise Deterministic Markov Process:

$$\dot{m} = r_m \chi - \gamma_m m \tag{37}$$

$$\dot{p} = r_p m - \gamma_p p \tag{38}$$

$$\chi : 0 \to 1 \text{ with rate } k_{on} \tag{39}$$

$$1 \to 0 \text{ with rate } k_{off} \tag{40}$$

3.3 Two-state model with auto-repression

To incorporate auto-repression into the two-state model, we allow the Her1 protein concentration to modulate the transcription rate, r_m ("amplitude regulation"), the OFF→ON rate, k_{on} ("frequency regulation"), the ON→OFF rate, k_{off} ("duration regulation"), or a combination thereof.

Amplitude regulation:

We model amplitude regulation via

$$r_m = r_{m,0} + r_{m,1} \frac{1}{K_D^n + p_\tau^n}. \tag{41}$$

where $p_\tau \equiv p(t-\tau)$ is the time-delayed protein concentration, taken at a time τ in the past.

The transcription rate has a basal rate, $r_{m,0}$, and a protein-dependent rate with prefactor $r_{m,1}$. The protein-dependence is described by a decreasing Hill function with coefficient, n, and scale K_D.

Because the timing of bursts is purely stochastic under this model, burst frequency must be high, such that each protein oscillation is made by many transcriptional bursts and it is the size of the bursts that is regulated. In fact, with $r_{m,0} = 0$, in the limit of infinitely fast bursts, this model reduces to the delay-differential equation model. To see this correspondence, note that the fraction

$$\frac{k_{on}}{k_{on} + k_{off}} \equiv P_{on} \tag{42}$$

is the steady state probability of the promoter being in the ON state. Thus, in the limit $k_{on} \to \infty$, $k_{off} \to \infty$, $k_{on}/(k_{on} + k_{off}) \to$ const., we have a separation of timescales between promoter switching and concentration dynamics and can replace the promoter state χ by it's steady state value p_{on}. Thus, the equation for m limits to

$$\dot{m} \to r_{m,1} P_{on} \frac{1}{K_D^n + p_\tau^n} - \gamma_m m, \tag{43}$$

recovering the delay differential equation. Accordingly, we found empirically that an explicit delay is required to obtain quasi-regular oscillations.

Frequency regulation:

We model frequency regulation via

$$k_{on} = k_{+,0} + k_{+,1} \frac{1}{K_D^n + p^n}. \tag{44}$$

In this model, Her1 protein decreases the rate at which the promoter switches from OFF to ON. We found empirically that an explicit delay was not required to obtain quasi-regular oscillations. This is likely because the extra delay needed for oscillations comes from the timing of individual bursts, which can be on the order of the oscillation period. For simplicity, in the main text we consider $k_{+,0} = 0$ and denote $k^+ \equiv k_{+,1}$.

Duration regulation:

We model duration regulation via

$$k_{off} = k_{-,0} + k_{-,1} \frac{p^n}{K_D^n + p^n}. \tag{45}$$

In this model, Her1 protein increases the rate at which the promoter switches from ON to OFF. Similar to frequency regulation, we found empirically that an explicit delay was not required to obtain quasi-regular oscillations. For simplicity, in the main text we consider $k_{-,0} = 0$ and denote $k^- \equiv k_{-,1}$.

3.4 Simulation details

We simulated this model using a hybrid Euler-Gillespie scheme[7]. In this scheme, mRNA and protein dynamics are deterministically integrated using the Euler method, while the promoter state evolves according to Gillespie's stochastic simulation algorithm. The key to this hybrid scheme is to constrain the time to next reaction such that the switching rates, which are functions of protein concentration, do not change appreciably while waiting for the next reaction to occur.

Specifically, the time to next reaction, τ, is computed to be

$$\tau = \min(\tau_G, \tau_{min}) \tag{46}$$

where τ_G is the interval obtained by Gillespie's algorithm given the current system state, and τ_{min} is the minimum time that is allowed to elapse such that the system does not appreciably

change. Several schemes for adaptively implementing this constraint exist, which make the algorithm accurate for arbitrary model parameters[7, 8]. However, here we are only interested in modeling the segmentation clock, for which half-lives of mRNA and protein species have been measured to be on the order of 10 minutes in zebrafish[2]. Thus, we use a fixed maximum jump time of $\tau_{min} = 1$ minute, over which the change in mRNA and protein concentrations will always be small. We used an Euler step size of $\Delta t = 0.01$ minutes.

3.5 Connection to other work

Our model contains as special cases models from various other works. In particular, Wang et al.[9] studied a protein-only version of the duration regulation model in the case of linear repression, $k_{off} = k_{-,1}p$, and found approximate analytic conditions for quasi-regular oscillations. However, the protein oscillations in this linear model are not as regular as experimentally-measured protein oscillations; we found that non-linearity appears to help achieve cleaner oscillations.

Work by Zinani et al. [10] considers a fully stochastic amplitude regulation model, using a high burst frequency of approximately 4 / minute. As we imaged every 1 minute in our experiments, we cannot rule in or out dynamics at that fast of a timescale.

The model of Lengyel et al. [11] does not assume bursting in the absence of auto-repression as our model allows, but demonstrates that a stochastic duration regulation-like mechanism, where Her1 proteins cooperatively shuts off transcription, can produce quasi-regular, burst-like oscillations. This model is similar to our model of duration regulation with no basal rate of bursting ($k_{-,0} = 0$) and a Hill coefficient that roughly equates to the number of Her1 binding sites in their model. Of note, like ours, their model requires a high level cooperativity to get realistic oscillations. Specifically, they consider a system of cooperative Her1 binding sites and require 10-20 binding sites to get regular oscillations. In our preliminary explorations, we similarly observed that a Hill coefficient of 10 or greater was required to get regular oscillations. Also like our model, the stochastic model of Lengyel et al. [11] does not require additional delay to achieve regular oscillations.

Finally, we note that there is a substantial body of theoretical work on models of bursting with feedback that is concerned with the distribution of mRNA and protein copy numbers [12]. The degree to which this distribution, even the time-dependent distribution [13], gives insight into the regularity of individual oscillations remains unclear.

References

[1] Hernan G. Garcia, Mikhail Tikhonov, Albert Lin, and Thomas Gregor. Quantitative Imaging of Transcription in Living Drosophila Embryos Links Polymerase Activity to Patterning. *Current Biology*, 23(21):2140–2145, November 2013.

[2] Julian Lewis. Autoinhibition with transcriptional delay: a simple mechanism for the zebrafish somitogenesis oscillator. *Current biology : CB*, 13(16):1398–1408, August 2003. Place: England.

[3] David S. Glass, Xiaofan Jin, and Ingmar H. Riedel-Kruse. Nonlinear delay differential equations and their application to modeling biological network motifs. *Nature Communications*, 12(1):1788, March 2021.

[4] B. C. Goodwin. Oscillatory behavior in enzymatic control processes. *Advances in enzyme regulation*, 3:425–438, 1965. Place: England.

[5] Alexis B Webb, Iván M Lengyel, David J Jörg, Guillaume Valentin, Frank Jülicher, Luis G Morelli, and Andrew C Oates. Persistence, period and precision of autonomous cellular oscillators from the zebrafish segmentation clock. *eLife*, 5:e08438, February 2016. Publisher: eLife Sciences Publications, Ltd.

[6] Arjun Raj, Charles S Peskin, Daniel Tranchina, Diana Y Vargas, and Sanjay Tyagi. Stochastic mRNA Synthesis in Mammalian Cells. *PLOS Biology*, 4(10):1–13, September 2006. Publisher: Public Library of Science.

[7] Eric L. Haseltine and James B. Rawlings. Approximate simulation of coupled fast and slow reactions for stochastic chemical kinetics. *The Journal of Chemical Physics*, 117(15):6959–6969, September 2002.

[8] Howard Salis and Yiannis Kaznessis. Accurate hybrid stochastic simulation of a system of coupled chemical or biochemical reactions. *The Journal of Chemical Physics*, 122(5):054103, January 2005.

[9] Jingkui Wang, Marc Lefranc, and Quentin Thommen. Stochastic oscillations induced by intrinsic fluctuations in a self-repressing gene. *Biophysical journal*, 107(10):2403–2416, November 2014. Place: United States.

[10] Oriana Q. H. Zinani, Kemal Keseroğlu, Supravat Dey, Ahmet Ay, Abhyudai Singh, and Ertuğrul M. Özbudak. Gene copy number and negative feedback differentially regulate transcriptional variability of segmentation clock genes. *iScience*, 25(7):104579, 2022.

[11] Iván M. Lengyel and Luis G. Morelli. Multiple binding sites for transcriptional repressors can produce regular bursting and enhance noise suppression. *Physical review. E*, 95(4-1):042412, April 2017. Place: United States.

[12] James Holehouse, Zhixing Cao, and Ramon Grima. Stochastic Modeling of Autoregulatory Genetic Feedback Loops: A Review and Comparative Study. *Biophysical journal*, 118(7):1517–1525, April 2020. Place: United States.

[13] Bingjie Wu, James Holehouse, Ramon Grima, and Chen Jia. Solving the time-dependent protein distributions for autoregulated bursty gene expression using spectral decomposition. Part I: Conventional models. *bioRxiv*, 2023. Publisher: Cold Spring Harbor Laboratory _eprint: https://www.biorxiv.org/content/early/2023/11/22/2023.11.21.568174.full.pdf.
